# Vsx2 Controls Eye Organogenesis and Retinal Progenitor Identity Via Homeodomain and Non-Homeodomain Residues Required for High Affinity DNA Binding

**DOI:** 10.1371/journal.pgen.1002924

**Published:** 2012-09-20

**Authors:** Changjiang Zou, Edward M. Levine

**Affiliations:** Department of Ophthalmology and Visual Sciences, John A. Moran Eye Center, University of Utah, Salt Lake City, Utah, United States of America; New York University, United States of America

## Abstract

The homeodomain and adjacent CVC domain in the *visual system homeobox* (VSX) proteins are conserved from nematodes to humans. Humans with missense mutations in these regions of *VSX2* have microphthalmia, suggesting both regions are critical for function. To assess this, we generated the corresponding mutations in mouse *Vsx2*. The homeodomain mutant protein lacked DNA binding activity and the knock-in mutant phenocopied the null mutant, *ocular retardation J*. The CVC mutant protein exhibited weakened DNA binding; and, although the corresponding knock-in allele was recessive, it unexpectedly caused the strongest phenotype, as indicated by severe microphthalmia and hyperpigmentation of the neural retina. This occurred through a cryptic transcriptional feedback loop involving the transcription factors *Mitf* and *Otx1* and the Cdk inhibitor *p27^Kip1^*. Our data suggest that the phenotypic severity of the CVC mutant depends on the weakened DNA binding activity elicited by the CVC mutation and a previously unknown protein interaction between *Vsx2* and its regulatory target *Mitf*. Our data also suggest that an essential function of the CVC domain is to assist the homeodomain in high-affinity DNA binding, which is required for eye organogenesis and unhindered execution of the retinal progenitor program in mammals. Finally, the genetic and phenotypic behaviors of the CVC mutation suggest it has the characteristics of a recessive neomorph, a rare type of genetic allele.

## Author Summary

Problems with the early development of the mammalian retina can cause congenital eye defects such as microphthalmia, in which the eye is dramatically smaller and functionally compromised. Severe microphthalmia is associated with mutations in the retinal-expressed *visual system homeobox 2* (Vsx2) gene, but how Vsx2 controls retinal development, and ultimately eye formation, has remained unclear. We assessed the impact of two missense mutations, discovered in humans, on Vsx2 function and eye development in mice. One mutation altered a highly conserved residue of the homeodomain, and the other altered a highly conserved residue in the CVC domain, a region of unresolved function. Both mutations impacted the DNA binding properties of the protein, although to differing extents. Likewise, both mutations caused microphthalmia and disruptions in retinal development, also to differing extents and by distinct mechanisms. Our data suggest that Vsx2 acts as a gatekeeper of the retinal gene expression program by preventing the activation of interfering or competing gene expression programs. We propose that the evolutionary stable association between the VSX-class homeodomain and CVC domain set the stage for Vsx2 or its archetype to assume a gatekeeper function for retinal development and ultimately eye organogenesis.

## Introduction

The homeodomain is a 60 amino acid DNA binding module composed of three alpha helices in a helix-turn-helix configuration. Homeodomain proteins are among the most numerous of transcription factors, second only to C2H2 zinc finger transcription factors in humans [1]. Structural studies of isolated homeodomains and site-directed mutants indicate that the properties needed for DNA binding are encoded within the homeodomain [2], [3], and two recent DNA binding screens of 168 mouse and 84 *Drosophila melanogaster* homeodomain proteins identified upwards of 16 amino acids occupying specific positions in the homeodomain that confer DNA binding site preferences and may define a general lexicon for predicting or rationally altering binding properties [4]–[6].

Many homeodomains, however, exhibit inherently low sequence specificity or weak binding affinity, characteristics inconsistent with their high degree of functional specificity *in vivo*. Solutions to this problem include the incorporation of additional DNA binding domains (e.g. Pou, Paired) or protein interaction domains that recruit additional DNA binding proteins (e.g. LIM) [7]–[10]. Other solutions do not incorporate modular domains, but rather utilize non-homeodomain residues or motifs to assist the homeodomain. The DNA binding capacity of several Hox homeodomains is enhanced by a cooperative interaction with PBC homeodomain proteins and is mediated by the hexapeptide/YPWM motif, a stretch of conserved hydrophobic residues near the N-terminus of the Hox homeodomain. This interaction not only increases the complexity of the target sequence since both proteins bind DNA, but it also enhances the DNA binding affinity of the Hox homeodomain [11]. The C-terminal tail in PBC proteins is a helical region adjacent to the C-terminus of the homeodomain that increases the homeodomain's DNA binding affinity, not by acting as a protein interaction motif or by directly contacting DNA, but through an intramolecular interaction that assists in properly positioning the third alpha helix (DNA recognition helix) into the major groove of its DNA binding site [12], [13].

It is unclear whether non-homeodomain motifs are commonly used to enhance homeodomain function. Predicting which non-homeodomain residues or motifs are required for homeodomain function is difficult because these relationships depend on subtle and highly specific differences among homeodomains and to specific structural conformations of DNA sequences that comprise binding sites [14]. However, the hexapeptide and C-terminal tail have two properties in common: they are positioned close to the homeodomain and are evolutionarily conserved in a non-modular fashion, meaning that they are only found in proteins with similar homeodomains.

**Figure 1:**
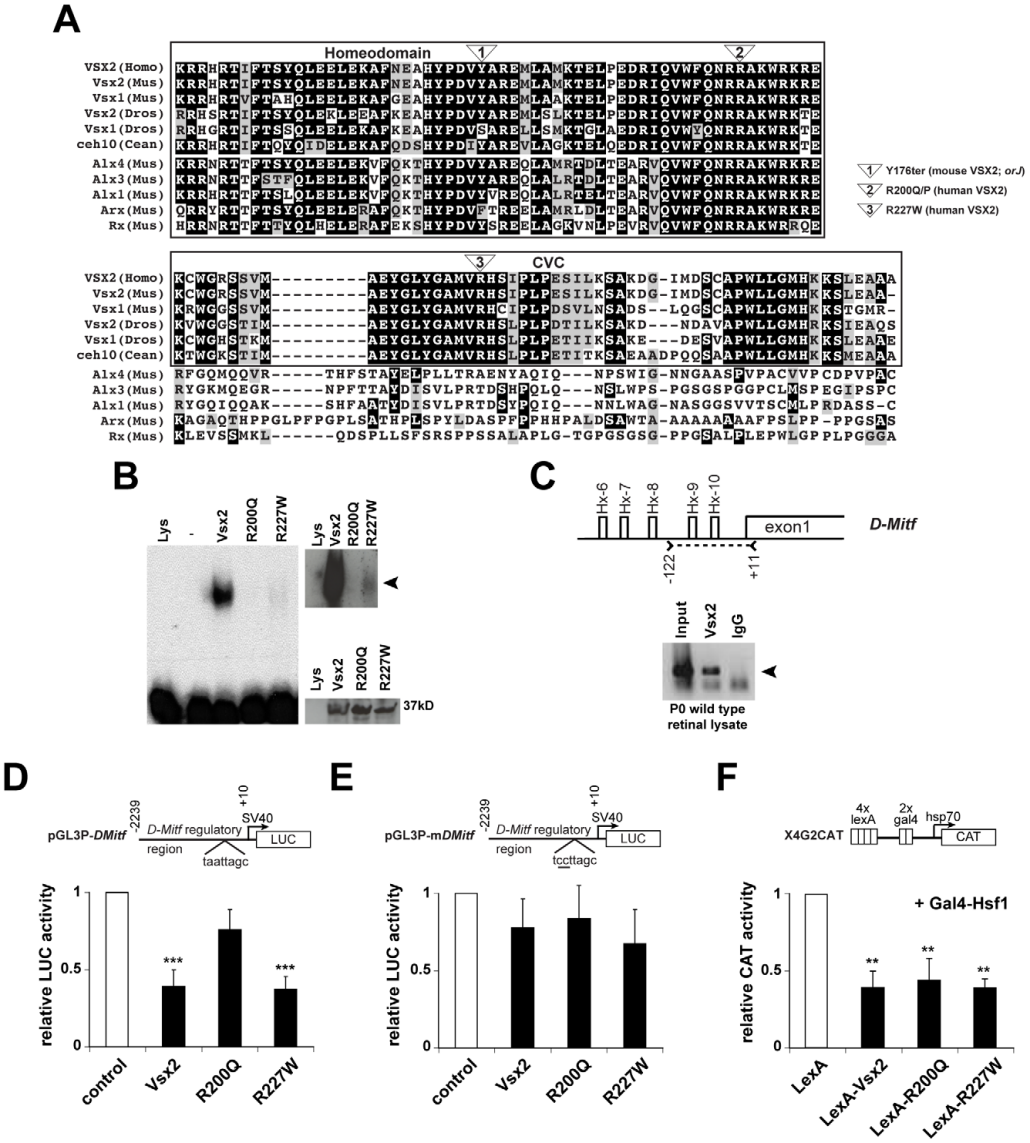
DNA binding and transcriptional activities of VSX2 and the VSX2^[R200Q]^ and VSX2^[R227W]^ variants. (A) ClustalW alignment of the homeodomain and adjacent 60 amino acids in select VSX orthologs and the most similar non-VSX proteins in mice. Only the VSX sequences have a discernable CVC domain. The positions of the *orJ*, *R200Q*, and *R227W* mutations are shown. (B) *Left panel:* EMSA with *in vitro* translated VSX2, VSX2^[R200Q]^, and VSX2^[R227W]^ proteins and [^32^]P-labeled P3 oligo (see Table S1 for sequence). *Top right panel:* Extended exposure reveals weak binding by VSX2^[R227W]^. *Bottom right panel:* Western blot of *in vitro* translated proteins with VSX2 antibody (Lys, control lysate; -, P3 probe only). (C) Schematic shows five putative Vsx2 binding sites (Hx-6 – Hx-10) in the proximal promoter region (∼0.3 kb) of *D-Mitf*. Carats and dashed line marks the region of PCR amplification in the ChIP assay shown below schematic (primer set 13; Table S1). Arrowhead points to sequence-verified ChIP product. (D) Luciferase assays in P0 primary retinal cells transfected with the indicated expression vectors (*x*-axis) and ∼2.2 kb of the *D-Mitf* promoter region (pGL3P-*DMitf*). (E) The Hx-9 site was mutated in pGL3P-m*DMitf* to eliminate DNA binding at that site. Reporter assays were normalized to empty vector controls (white bars). (F) CAT assays in HEK293 cells transfected with the X4G2CAT reporter and VSX2 variants fused to the LexA DNA binding domain. Gal4-Hsf1 was included to stimulate high basal reporter activity [34]. ** P≤0.01; *** P≤0.001.

Another group of homeodomain proteins with a conserved, non-modular motif adjacent to the homeodomain are in the *visual system homeobox* family (VSX; also referred to as Prd-L:CVC or CVC paired like). These include Vsx1 and Vsx2 (formerly Chx10) in vertebrates and *D. melanogaster* and ceh-10 in *Ceanorhabditis elegans*. VSX genes belong to the larger paired-like homeodomain class that include Rx, Arx, and Alx genes [10], but are unique in that they encode a region of approximately 60 amino acids extending from the C-terminus of the homeodomain and named the CVC domain for the genes in which it was initially discovered (Figure 1A) [15]–[17]. Genetic data suggest the CVC domain is essential for VSX function. In *C. elegans*, two missense mutations in the ceh-10 CVC domain cause embryonic lethality and neuronal differentiation defects similar in severity and timing to those elicited by nonsense mutations [18], [19]. In humans, missense mutations in the *VSX1* and *VSX2* CVC domains are linked to ocular abnormalities and disease [20]–[25]. While the pathogenicity of the *VSX1* CVC variants is unclear [26], [27], evidence for *VSX2* is strong. In two consanguineous families, the arginine at position 227, an invariant residue among VSX genes and part of the CVC domain, is substituted with tryptophan (Figure 1A) and this mutation segregates in a homozygous fashion with non-syndromic congenital bilateral microphthalmia (small eye; [20], [24]). A recent case study identified a new missense mutation in the CVC domain (alanine substituted for glycine at position 223), which also segregates in a homozygous fashion with microphthalmia [25]. These mutations are likely to have a profound effect on protein function since microphthalmia occurs in humans with other mutations in *VSX2*, most notably missense mutations in the homeodomain which substitute glutamine or proline for arginine at position 200 (Figure 1A; [24], [28], [29]. Vsx2-dependent microphthalmia also occurs in homozygous *ocular retardation J* mice (MGI symbol: *orJ*), which harbor a nonsense mutation in the homeodomain (Figure 1A). VSX2 protein is not detected from the *orJ* allele (this study; [30]), and this allele is therefore considered to be a null. Small eye phenotypes are also observed in zebrafish subjected to Vsx2 mRNA knockdown [31]–[33].

Addressing whether the CVC domain assists in homeodomain function is complicated by the likelihood that the CVC domain has multiple functions. Its deletion in VSX2 altered DNA binding and transcriptional properties although it is unclear whether these changes were interdependent, and whether they were specific to the CVC domain because other regions were also removed [34]. Its deletion in Vsx1 reduced polyubiquitination suggesting a role in regulating protein stability [35]. Because deleting the entire CVC domain could lead to pleiotropic effects, another approach to identify functional requirements of the CVC domain and its relationship with the homeodomain is to study the effects of the missense mutations on protein function and eye development.

In this study, we generated the homeodomain mutation *R200Q* and CVC domain mutation *R227W* in the mouse *Vsx2* ortholog and compared their functional properties. A predominant effect of these mutations is to reduce homeodomain-dependent DNA binding but to different degrees. Since Vsx2 regulates eye size and retinal development, we generated knock-in mice and compared their phenotypes to the *orJ* mouse. Molecular and genetic analyses enabled us to identify the transcriptional circuits driving the phenotypes caused by each mutation. Our data support the model that the proper execution of mammalian eye organogenesis and retinal development is built upon high affinity DNA binding by Vsx2, which is dependent on both the homeodomain and CVC domain. We also provide evidence suggesting that Vsx2 regulates one of its key targets, *microphthalmia-associated transcription factor* (Mitf) by two mechanisms; direct transcriptional repression and protein∶protein interaction. Both mechanisms may be employed to prevent activation of aberrant gene expression programs that interfere with the execution of the developmental program in retinal progenitor cells (RPCs).

## Results

### The *R200Q* and *R227W* mutations alter the DNA binding affinity of VSX2 protein, but not its ability to repress transcription

We compared the DNA binding properties of in vitro translated VSX2 and the VSX2^[R200Q]^ and VSX2^[R227W]^ variants using an oligonucleotide containing a high affinity Vsx2 binding site by electrophoretic mobility shift assays (EMSA). Consistent with previous studies [28], [32], robust DNA binding was observed for VSX2 whereas binding was not detected with VSX2^[R200Q]^ (Figure 1B, left panel). VSX2^[R227W]^ binding was detectable but weak, and is more visible with a longer exposure time (Figure 1B, top right panel). The reduced DNA binding properties were not due to variations in protein expression (Figure 1B, bottom right panel) or to alterations in the helical organization of the homeodomain as assessed with secondary structure prediction software (http://us.expasy.org; data not shown). These observations indicate that the arginines at positions 200 and 227 are required for high affinity DNA binding.

Of the known candidate targets of Vsx2-mediated transcriptional regulation [32], [36]–[38], the basic helix-loop-helix/leucine zipper (bhlh/zip) gene *Mitf* is of high importance because its increased expression in the *orJ* retina contributes to the mutant phenotype [39]–[42]. Regulation of the *Mitf* locus is complex. Nine promoters have been identified and each produces an RNA transcript with a distinct first exon, several with limited protein-coding information. As a result, multiple *Mitf* isoforms on the RNA and protein levels are possible, although most if not all isoforms contain the domains and motifs needed for transcription factor activity (see Figure 1 in [39] for a detailed illustration of the mouse *Mitf* locus and gene products). Depending on the cell type, *Mitf* isoforms are expressed in different combinations, indicating that promoter utilization is context-dependent [43]. D-Mitf is one of the isoforms upregulated in the *orJ* retina and has at least 10 putative Vsx2 binding sites within 1 kb upstream of its transcriptional start site (Hx-1 – Hx-10; [39]). Chromatin immunoprecipitation (ChIP) assays revealed VSX2 binding in the vicinity of the Hx-1 – Hx-3 sites, approximately 0.8 kb upstream of the *D-Mitf* transcriptional start site [39]. We found that VSX2 bound to chromatin in the vicinity of the Hx-6 – Hx-10 sites, less than 0.3 kb upstream *D-Mitf* transcriptional start site (Figure 1C). Based on these findings, the *D-Mitf* promoter is likely to be a direct target of Vsx2-mediated transcriptional repression.

To test this further, transcriptional reporter assays were done with a construct containing ∼2.2 kb of the *D-Mitf* promoter region and SV40 early promoter driving a luciferase reporter (pGL3P-*DMitf*) and constructs for expressing VSX2, VSX2^[R200Q]^, or VSX2^[R227W]^ proteins in P0 wild-type retinal cells. Inclusion of the SV40 promoter was necessary for reliable basal activity reporter activity. Consistent with our results for the *Vsx1* promoter [32], VSX2 repressed reporter activity and repression mediated by VSX2^[R200Q]^ was diminished (Figure 1D). Like Vsx2, VSX2^[R227W]^ also repressed reporter activity and required the presence of the Hx-9 site (Figure 1D, 1E). Similar results were obtained in HEK293 cells (data not shown), indicating that repression of reporter activity was not dependent on additional factors exclusive to retinal cells. The SV40 promoter was not used in these and all other reporter assays in HEK293 cells because it was not required for basal reporter activity. To determine if the mutations interfered with repressor function in addition to DNA binding, the LexA DNA binding domain was fused to the Vsx2 variants and reporter activity was determined in HEK293 cells with X4G2CAT, a choloramphenicol acetyltransferase (CAT) reporter containing multimerized LexA binding sites [34]. In this assay, each of the LexA fusions repressed CAT reporter activity to similar extents (Figure 1F). Thus, the repression mediated by VSX2 and VSX2^[R227W]^ depended on DNA binding and the R200 and R227 residues were not required for repressor activity.

### Generation and characterization of *Vsx2^R200Q^* and *Vsx2^R227W^* knock-in mice

If DNA binding is essential for Vsx2 function, then the VSX2^[R200Q]^ protein should be a functional null and the VSX2^[R227W]^ protein should retain some degree of Vsx2 function. If true, then the simplest predictions for mice with these mutations are that homozygous *Vsx2^R200Q^* mutants (*R200Q*) should phenocopy *orJ* mutants and homozygous *Vsx2^R227W^* mutants (*R227W*) should exhibit a less severe or hypomorphic phenotype. It is also possible that these alleles could exhibit dominant negative activity if Vsx2 activity requires dimerization, similar to DNA binding mutations in the homeodomain protein Pitx2, which are linked to Axenfeld-Rieger syndrome [44],[45]. We tested these predictions by generating *R200Q* and *R227W* knock-in mice by homologous recombination using the ACN targeting vector, which removed all gene targeting elements when the alleles were transmitted through the male germline (Text S1; Figure S1; [46]). The only foreign DNA retained was a 34 base pair sequence containing a remnant lox-p site in intron 3 (Figure S1). Germline transmission was achieved (Figure S1) and the mutants used for this study were established in the129sv genetic background to minimize strain-dependent modifier effects [40], [41], [47], [48].

**Figure 2:**
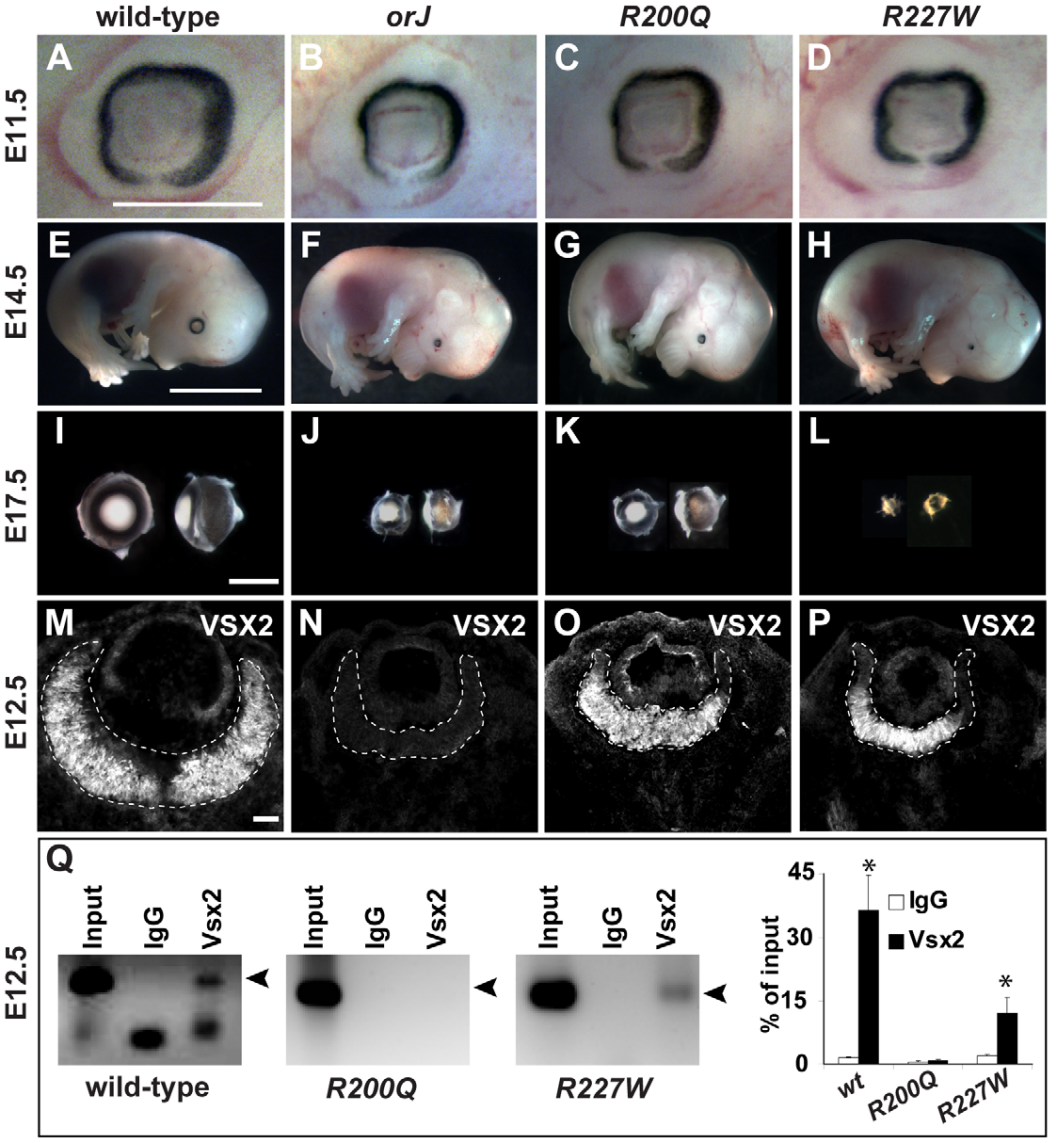
The *R200Q* and *R227W* mutations cause non-syndromic congenital microphthalmia. (A–D) Mice homozygous for the *orJ*, *R200Q*, and *R227W* alleles had smaller eyes than wild-type by E11.5. (E–H) At E14.5, overall embryonic development was unaffected in the mutants, but the failure of the mutant eyes to keep pace with the growth of the wild-type eye was evident. Eye growth in the *R227W* mutant also failed to keep pace with the *orJ* and *R200Q* mutants. (I–L) Dissected E17.5 eyes (right eyes rotated 90°) show similar reductions in eye size in *orJ* and *R200Q* homozygotes whereas the reduction in eye size of *R227W* homozygotes was the most severe. (M–P) VSX2 immunohistochemistry in E12.5 retinas. VSX2 protein was not detected in the *orJ* retina, confirming it as an expression null. VSX2^[R200Q]^ and VSX2^[R227W]^ were expressed similarly to VSX2^[wt]^, although to a reduced extent in peripheral retina. Dashed lines bound retinas. (Q) ChIP assays with VSX2 antibody reacted with E12.5 native chromatin lysates from wild-type, *R200Q*, and *R227W* retinas and amplified using *D-Mitf* primer set 13 (Table S1). Arrowhead denotes amplification product. Graph shows quantification results of ChIP-qPCR. Scale bars: 0.5 mm (E11.5); 5 mm (E14.5); 1 mm (E17.5).

As in humans, *R200Q* and *R227W* mice were microphthalmic, which was apparent by E11.5 and became progressively more severe as development continued (Figure 2A–2L). Relative eye size in *orJ* and *R200Q* mutants were similar, but surprisingly, *R227W* mutants exhibited smaller eyes at E14.5 and beyond (Figure 2E–2L). In contrast to the lack of VSX2 protein in *orJ* RPCs, both knock-in mutants expressed VSX2 protein in a manner similar to wild-type, suggesting that changes in expression or nuclear localization were not causing the phenotypes (Figure 2M–2P; Figure S2A). Consistent with the EMSA data, ChIP assays with E12.5 retinal lysates showed that VSX2^[R200Q]^ protein was not detectable at the *D-Mitf* promoter whereas VSX2^[R227W]^ was bound although to a lesser extent than VSX2 (Figure 2Q).

**Figure 3:**
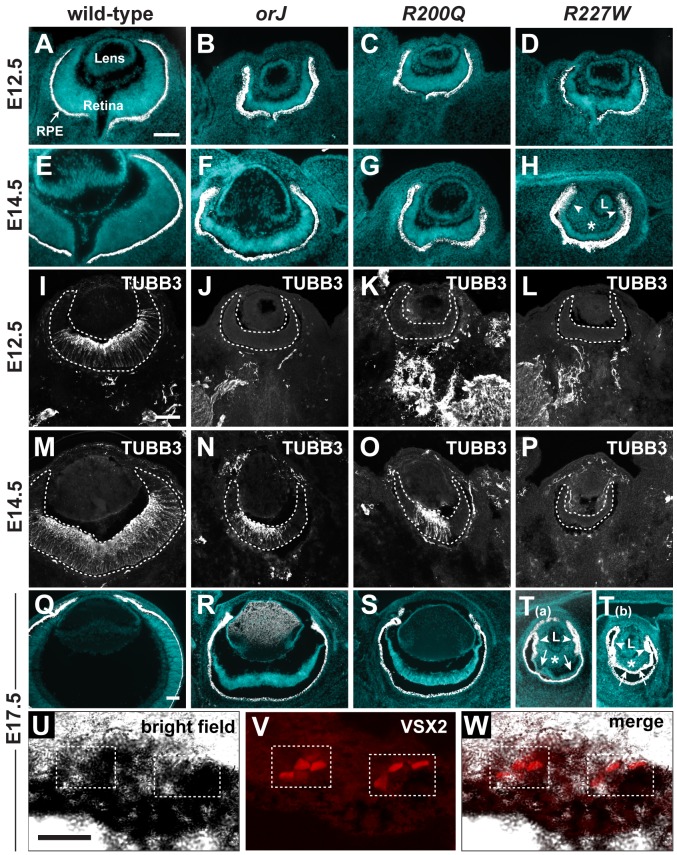
Ocular histology and neurogenesis in *Vsx2* mutants. (A–H) Merged images of cryosections showing DAPI staining (blue) and melanogenic pigmentation (white) for each of the indicated genotypes and ages. Arrowheads in H point to aberrant pigmentation in peripheral retina asterisk denotes ectopic periocular mesenchyme (POM) in vitreal cavity. (I–P) Expression patterns of the neuronal differentiation marker class III β-Tubulin (TUBB3). Neurogenesis lagged behind wild-type and to a similar extent in the *orJ* and *R200Q* retinas, but did not initiate in the *R227W* retina. (Q–T) Merged images of cryosections showing DAPI staining (blue) and melanogenic pigmentation (white) for each of the indicated genotypes at E17.5. The *R227W* retina was aberrantly pigmented, either partially (T_(a)_) or completely (T_(b)_). Arrowheads in T_(a)_ and T_(b)_ point to aberrant pigmentation in peripheral retina, arrows to central retinal regions, and asterisks to ectopic pigmentation in vitreal cavity. (U–W) Pigmented cells expressing VSX2^[R227W]^ were detected in pigmented retinal region. L, lens; RPE, retinal pigment epithelium. Scale bars: 100 µm (A–T), 20 µm (U–W).

Histological analysis revealed that mutant eyes had a smaller lens, thickened retinal pigment epithelium (RPE), and a thinner retina compared to wild-type (Figure 3A–3H). Whereas the *orJ* and *R200Q* eyes were similar in appearance, *R227W* eyes were more severely affected as indicated by an even smaller lens, thinner retina, and an infiltration of mesenchymal cells into the vitreal chamber (Figure 3H, asterisks). The smaller retina in the *orJ* mouse is correlated with a reduction in RPC proliferation [30], [47], [49]–[53] and this was likely the case in the *R200Q* and *R227W* retinas as both mutants showed reduced phosphorylated Histone H3 expression (Figure S2B). Consistent with this, VSX2^[R200Q]^ or VSX2^[R227W]^ overexpression in cultured *orJ* retinal cells was not sufficient to promote proliferation (Figure S2C).

The onset of neurogenesis occurs by E11.5 in the central retina and spreads as a wave toward the peripheral retina. This was delayed by 1–2 days in *orJ* and *R200Q* retinas and was not observed at all in the embryonic *R227W* retina (Figure 3I–3P; Figure S3; [47], [52]). The lack of detectable neurogenesis suggested that *R227W* RPCs underwent a fundamental change in their developmental potential that differed from the other mutants.

Optic cup morphogenesis initiates at approximately E9.5, soon after onset of Vsx2 expression [17], [54]. The Vsx2 expression domain marks the interior layer of the optic cup and gives rise to the retina. Under normal conditions, melanogenic pigmentation does not occur in the retina. In contrast, pigmentation and thinning of the epithelium is pronounced in the peripheral retina of *orJ* mice, and lineage analysis suggests these pigmented cells arose from retinal-specified progenitor cells [40]. Pigmentation of the central *orJ* retina is rare in the 129svj genetic background, but is fairly prevalent in mixed 129svj:C57Bl6 mice owing to uncharacterized genetic modifiers [40], [41]. Similar background-dependent effects were observed for the *R200Q* retina (data not shown). Surprisingly, pigmentation was much more extensive in the *R227W* retina of mice with the 129sv genetic background (Figure 3Q–3T). This occurred in a progressive manner, which started in the peripheral retina at E14.5 and extended centrally to occupy most or all of the retina by E17.5 (Figure 3H, 3T_(a)_, 3T_(b)_). VSX2^[R227W]^ protein was detected in some pigmented cells at E17.5, which indicated that as in the *orJ* retina, the ectopically pigmented cells arose from RPCs (Figure 3U–3W). In contrast, VSX2^[R200Q]^ protein remained expressed in RPCs at E17.5 (data not shown). These data suggest that the pigmentation of the *R227W* retina was a direct consequence of the mutant allele.

**Figure 4:**
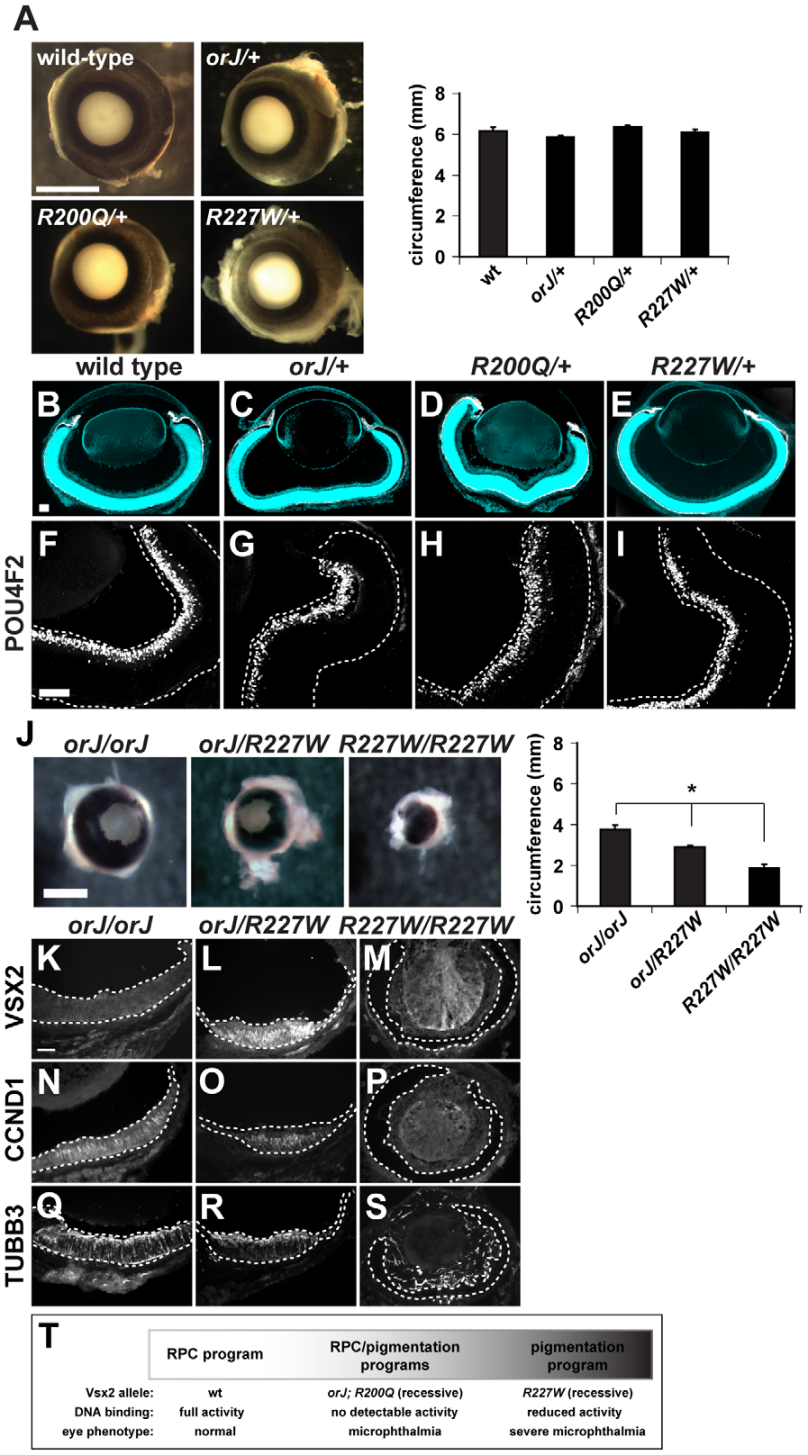
The *R200Q* and *R227W* alleles and proteins do not exhibit dominant behavior. (A) Wild-type, *orJ/+*, *R200Q/+*, and *R227W/+* eyes were indistinguishable at P0. No significant differences in eye circumferences were detected. (B–E) Merged images of cryosections showing DAPI staining (blue) and melanogenic pigmentation (white) for each of the indicated genotypes at P0. (F–I) Expression of the retinal ganglion cell marker POU4F2 in wild-type or *Vsx2* heterozygous retinas. (J) Eye circumference of *orJ/R227W* heterozygotes was intermediate to *orJ* and *R227W* homozygotes. (K–S) Expression of VSX2, CCND1, and TUBB3 in *orJ*, *orJ/R227W*, and *R227W* retinas at P0. VSX2 was detected in the *orJ/R227W* retina only. CCND1 and TUBB3 were detected in *orJ* or *orJ/R227W* retinas but not in *R227W* pigmented retina. (T) Genotype-phenotype correlation of *Vsx2* alleles arranged by retinal phenotype. * P≤0.05 Scale bars: 1 mm (A, J); 100 µm (B–I, K–S).

In agreement with our *in vitro* data, the *R200Q* and *orJ* phenotypes were similar and support the hypothesis that homeodomain-dependent DNA binding is critical for Vsx2 function. That the *R227W* phenotype was more severe was unexpected since the VSX2^[R227W]^ protein retained properties associated with transcriptional regulation. Our *in vivo* and *in vitro* data are consistent in that they indicate the VSX2^[R227W]^ protein was functional, but they differ because the *in vivo* phenotype indicated that the *R227W* allele was not hypomorphic. One possibility for this discrepancy is that the VSX2^[R227W]^ protein acquired a novel activity not revealed by the *in vitro* assays. If this were true, then the R227W allele should be dominant or semi-dominant. This was not the case, however, since eye size, circumference, and retinal histology in *R227W/+* mice were indistinguishable between wild-type, *orJ/+* or *R200Q/+* mice (Figure 4A–4I; Figure S4). Furthermore, hemizygous *R227W/orJ* mice exhibited an intermediate phenotype compared to the *orJ* and *R227W* homozygotes (Figure 4J–4S), which suggested that the failure of the *R227W* allele to compete with the wild-type allele was not due to reduced expression of the mutant protein. Finally, overexpression of VSX2, VSX2^[R200Q]^, or VSX2^[R227W]^ in newborn wild-type retinal primary cells had minimal effects on proliferation (Figure S2D). These data revealed that even if the VSX2^[R227W]^ protein acquired a novel activity, it is not sufficient to interfere with wild-type Vsx2 function. Thus, even though the *R227W* phenotype surpasses the null in severity, the allele displayed recessive behavior, consistent with what is observed in humans. Additionally, the recessive nature of the *R200Q* and *R227W* alleles suggest that Vsx2 does not require dimerization for function.

The data presented thus far best fits the genotype-phenotype correlation shown in Figure 4T. In wild-type mice, the RPC program driving retinal development progressed normally and correlated with unhindered DNA binding activity by Vsx2. In *orJ* and *R200Q* mice, the RPC program progressed in a suboptimal manner and the RPCs were biased, but not necessarily committed, to expressing a pigmentation program. The *orJ* and *R200Q* alleles were recessive and lacked Vsx2-dependent DNA binding activity. In the case of *orJ*, this is because the protein was not present, and in the case of *R200Q* because the DNA binding activity was specifically disrupted. In *R227W* mice, the RPC program initiated, but ultimately failed and was followed by robust expression of a pigmentation program. As with *orJ* and *R200Q*, the *R227W* allele was recessive, but the protein retained DNA binding activity, albeit weakened compared to wild-type.

### Multiple regulatory changes at the *Mitf* locus are associated with pigmentation in the *R227W* retina

**Figure 5:**
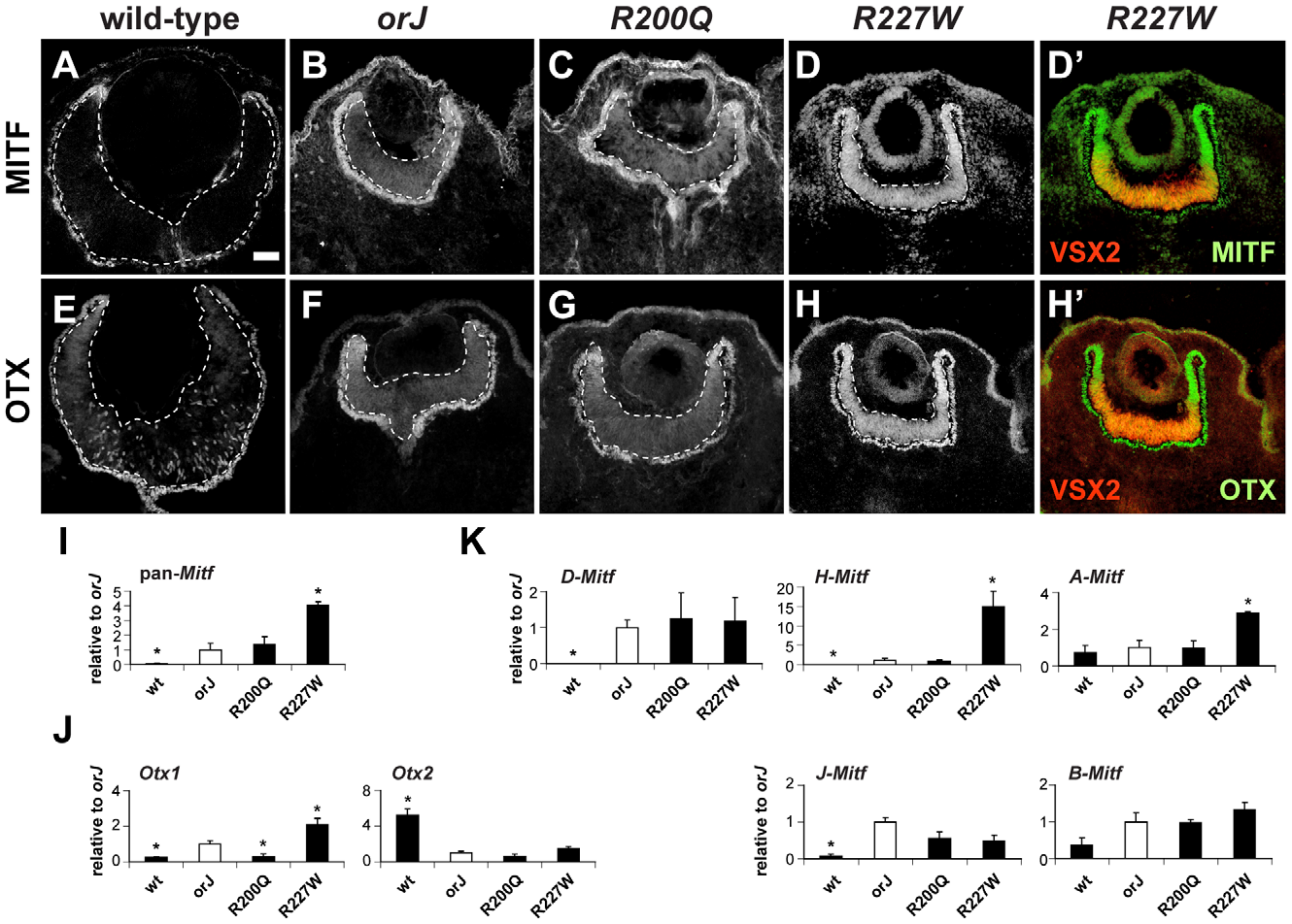
Phenotypic severity correlates with the expression levels of Mitf and Otx1. (A–D) MITF expression at E12.5 for the indicated genotypes. The *R227W* retina expressed MITF at much higher levels compared to the *orJ* and *R200Q* mutants. (D′) Merged images of VSX2^[R227W]^ (red) and MITF (green) shows overlap in expression. The lack of VSX2^[R227W]^ expression in the peripheral retina corresponded to the highest levels of MITF. (E–H) OTX expression at E12.5 for the indicated genotypes. OTX expression was highest in the *R227W* retina. (H′) Merged images of VSX2^[R227W]^ (red) and OTX (green). Like MITF, OTX expression was highest in regions lacking VSX2^[R227W]^. MITF and OTX were also expressed in RPE (outside lower dashed lines). (I–K) Relative mRNA expression levels of pan-*Mitf* (I), *Otx1* and *Otx2* (J), and the *D-*, *H-*, *A-*, *J-*, and *B*-*Mitf* isoforms (K) in E12.5 retinas of the indicated genotypes as determined by qRT-PCR. Samples were normalized to the expression level for each transcript in the *orJ* retina (white bars). * P≤0.05 Scale bar: 50 µm.

Mitf is initially expressed throughout the optic neuroepithelium at the optic vesicle stage and is downregulated in the presumptive retinal domain soon after Vsx2 is expressed [54]–[56]. Since Mitf is a key regulator of the genetic pathways that drive melanogenic pigmentation, we suspected that Mitf expression levels would correlate with the degree of pigmentation in the mutants. In *orJ* and *R200Q* mice, MITF expression at E12.5 was modestly elevated in the central retina and highest in the peripheral retina where pigmentation was most prevalent (Figure 5A–5C). In contrast, MITF was highly expressed throughout the R227W retina (Figure 5D). We also examined the expression of the orthodenticle-related homeodomain proteins OTX1 and OTX2 (OTX) since they are also required for the pigmentation program in the RPE and can directly regulate *Mitf* expression [57]–[60]. Similar to MITF, OTX expression was modestly increased in the *orJ* and *R200Q* central retinas but was highly expressed throughout the *R227W* retina with the most notable increases in the periphery (Figure 5E–5H; negative control staining shown in Figure S6C). Interestingly, VSX2 was downregulated in the peripheral retina, which correlated with the highest expression levels of MITF and OTX (Figure 5D′, 5H′) and where pigmentation was most apparent at E14.5 (Figure 3H), which suggested a change in fate for these cells. The scattered OTX expression in the wild-type retina is linked to the production of postmitotic Otx2^+^ cone photoreceptor precursors [61], which were not observed in the mutants, consistent with the delay or absence of neurogenesis.

To determine if changes in mRNA levels could account for the differences in MITF and OTX expression, we performed quantitative real-time reverse-transcription PCR (qRT-PCR) on total RNA lysates from E12.5 retinas. Using primers that recognize all Mitf isoforms (pan-*Mitf*), we found that compared to the *orJ* retina, wild-type had significantly less expression, *R200Q* was similar, and *R227W* had significantly more expression (Figure 5I), all of which is consistent with the immunohistochemical data. We also used gene specific primers for *Otx1* and *Otx2* to determine their relative expression levels in each genotype (Figure 5J). Compared to *orJ*, *Otx1* expression was lower in wild-type and *R200Q* and higher in the *R227W* retina. All of the mutants expressed *Otx2* at lower levels than wild-type. These data indicated that the increase in OTX protein expression was specific to *Otx1*. Furthermore, the low level of *Otx1* in the *R200Q* retina revealed a difference with the *orJ* retina, which was unexpected in light of the overall similarities in the cellular and tissue phenotypes of the two mutants.

The *A*, *D*, *H*, and *J* isoforms of *Mitf* are expressed in the embryonic retinal pigment epithelium (RPE) with *A*, *D*, and *H* being the most abundant [39]. These isoforms are also detected in the retina at much reduced levels with the exception of *A*, which is expressed at a level comparable to the RPE [39]. We performed qRT-PCR of E12.5 retinal RNA to determine the isoform-profile in the different genotypes (Figure 5K). We found that *D* and *H* were higher in the *orJ* retina than in wild-type, consistent with previous findings [39]. We also observed a significant increase in *J*, an upward trend in *B*, and no change in *A*. In general, the isoform expression profile in the *R200Q* retina was similar to *orJ*. In the *R227W* retina, however, only *A* and *H* exhibited higher expression compared to *orJ* and *R200Q*. These data suggested the increased *Mitf* expression in the *R227W* retina was due to novel changes in the transcriptional regulation of the *A* and *H* isoforms.

### Periocular mesenchyme (POM) promotes *A-Mitf* expression, but *H-Mitf* is the isoform that most likely accounts for the unique increase in the overall Mitf level in the *R227W* retina

**Figure 6:**
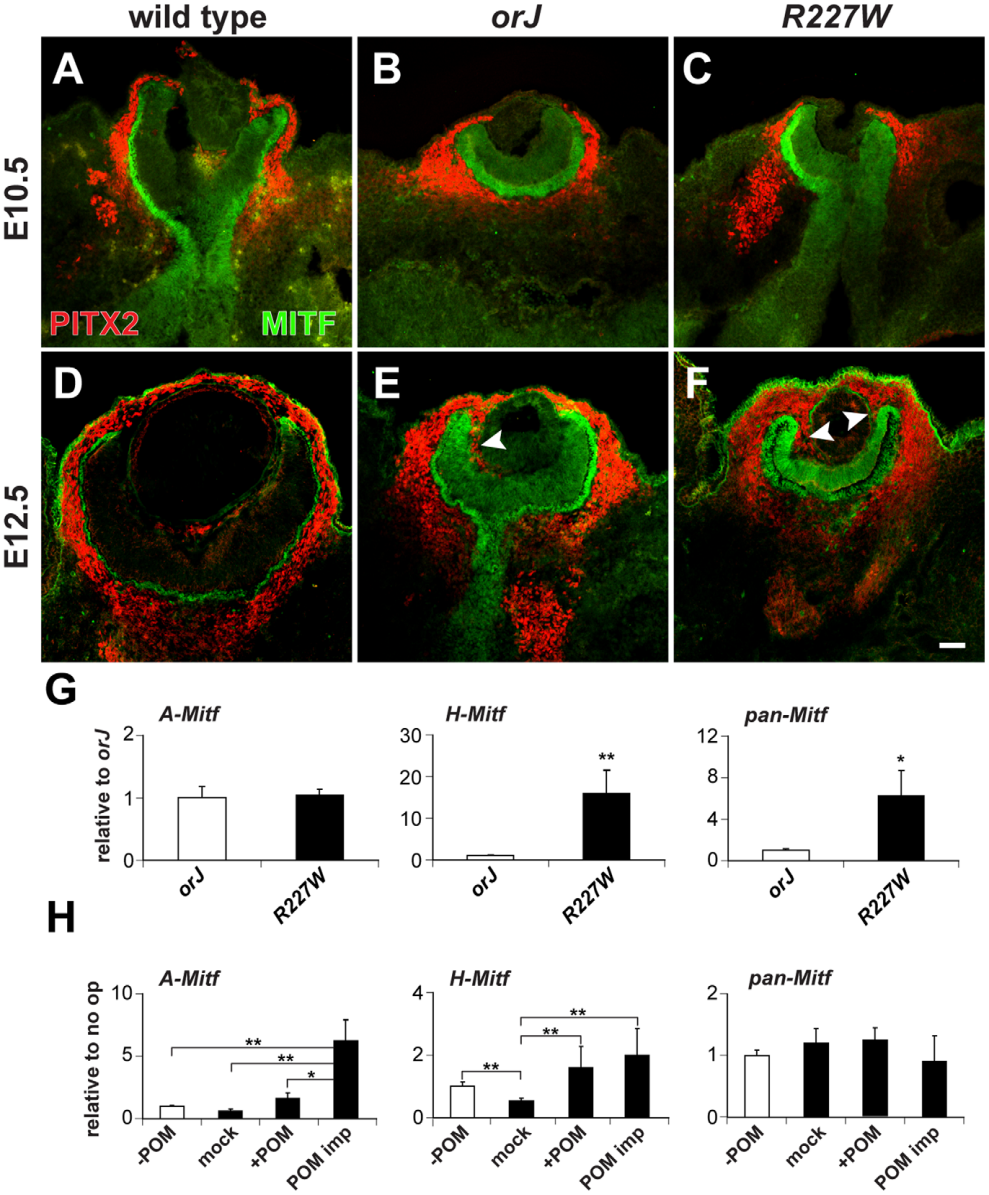
Influence of POM on Mitf expression in the *R227W* retina. (A–F) PITX2 (red) and MITF (green) expression at E10.5 (A–C) and E12.5 (D–F). Limited PITX2^+^ cells were detected in the vitreal chamber (between retina and lens) of wild-type eyes. PITX2^+^ cells were not detected in the vitreal chamber of mutant eyes at E10.5, but were abundant by E12.5 and continuous with POM at the retinal periphery (arrowheads). MITF expression levels were modestly upregulated at E10.5 in the mutant retinas and were clearly elevated by E12.5. (G) Relative expression levels of *A-Mitf*, *H-Mitf*, and pan-*Mitf* in E10.5 whole retina and lens explants cultured for 48 hr. *R227W* expression levels were normalized to *orJ*. Invasion of POM into the vitreal chamber did not occur in these cultures (data not shown). (H) Relative expression levels of *A-Mitf*, *H-Mitf*, and pan-*Mitf* in physically manipulated E10.5 *R227W* whole retina and lens explants cultured for 48 hr after the following manipulations: “−POM” (retina and lens only); “mock” (retina partially separated from lens); “+POM” (retina partially separated from lens with surrounding POM intact); “POM imp” (retina partially separated from lens and POM implanted into vitreal cavity). Expression levels were normalized to the “−POM” condition. * P≤0.05; ** P≤0.01 Scale bar: 50 µm.

The POM is composed of neural crest and mesoderm-derived progenitors that express *Pitx2* and contribute to the formation of ocular structures such as the sclera, choroid, and cornea [62]–[64]. In chick, the POM contains an Activin-like factor that promotes *Mitf* expression in the nascent RPE domain of the optic vesicle [65]. Interestingly, the most profound changes with respect to tissue morphology, pigmentation, and upregulation of Mitf and Otx1 expression corresponded to regions of the neural retina that were juxtaposed to POM that invaded the vitreal chamber from the retinal peripheral margin after E10.5 (Figure 6A–6F). This was most pronounced in the *R227W* retina in which the entire vitreal chamber fills with PITX2 positive cells by E17.5 (Figure S5A). This was highly unusual since very few mesenchymal cells normally migrate into the vitreal cavity [63].

To determine whether the POM was responsible for the elevated expression levels of the *A-* or *H-Mitf* isoforms in the *R227W* retina, we cultured retinal explants from E10.5 *orJ* and *R227W* embryos with the lens attached but POM and RPE removed for 48 hr and measured transcript levels. Infiltration of POM into the vitreal chamber was not detectable at E10.5 and we found no evidence of POM invasion after 48 hr in culture (data not shown). Under these conditions, *A-Mitf* was unchanged between the two mutants whereas *H-Mitf* was significantly higher in the *R227W* mutant (Figure 6G). To further test the influence of POM, we cultured E10.5 *R227W* explants containing whole retina and lens in the presence or absence of POM in four different conditions: POM removed (- POM); POM removed with vitreal space physically exposed (mock); retention of POM at the anterior pole (+POM); or POM implanted into the vitreal chamber (POM imp). The POM implant was the only condition in which *A-Mitf* expression was enhanced further (Figure 6H, left graph). Although the increase in *H-Mitf* expression was significant in the presence of POM (Figure 6H, middle graph), the magnitude of the change was small (note the difference in scale of the *y*-axes for the middle graphs in Figure 6G and 6H). The effects of the POM implant on the *D-* and *J-* isoforms were not significant (Figure S5B). These data suggested that the elevated level of *A-Mitf* was under the influence of signals from the POM whereas *H-Mitf* expression was largely independent of POM-derived signals. Since the high level of pan-*Mitf* expression also did not depend on the presence of POM (Figure 6H, right graph), *H-Mitf* appears to be the mRNA isoform primarily responsible for the elevated pan-*Mitf* level in the *R227W* retina and its regulation is likely to be cell-autonomous.

### Dominant-negative *Mitf* reveals a positive feedback loop for driving high Mitf expression and pigmentation in the *R227W* retina

**Figure 7:**
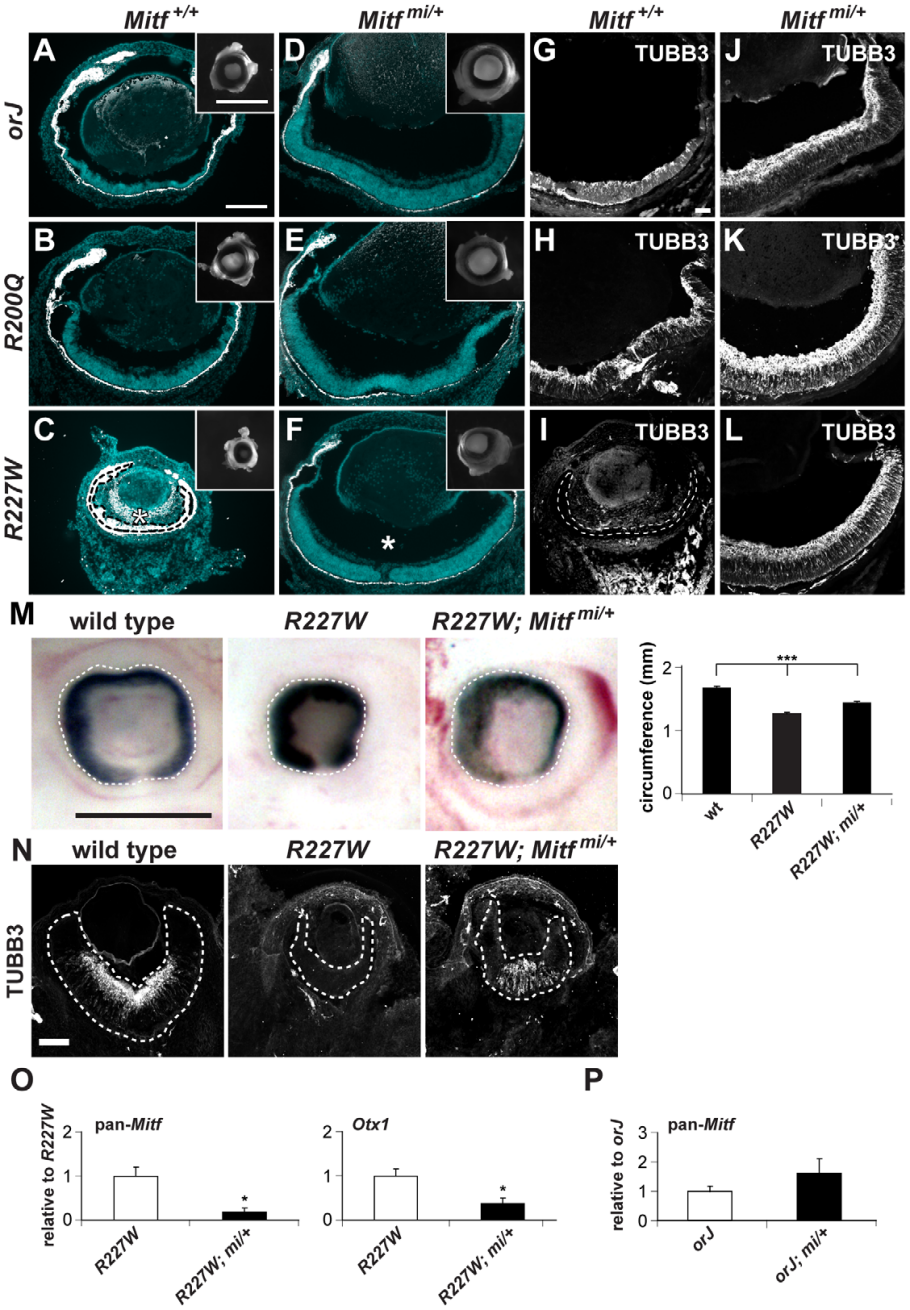
The dominant negative allele *Mitf^mi^* restores retinal development in the *Vsx2* mutants. (A–F) Merged images of cryosections showing DAPI staining (blue) and melanogenic pigmentation (white) in P0 *orj*, *R200Q*, and *R227W* mice that were *Mitf* wild-type (*Mitf^+/+^*; A–C) and *mi* heterozygous (*Mitf^mi/+^*; D–F). Insets show whole eyes. The retina in C was completely transformed into pigmented tissue (bounded by dashed line) and ectopic POM was partially pigmented (asterisk). Eye size and retinal histology were restored to a comparable degree in all *Vsx2*, *mi* compound mutants (D–F). Also notable in the *R227W*, *mi* compound mutant was the lack of POM in the vitreal chamber (asterisk in F). (G–L) TUBB3 staining at P0. In all cases, lamination patterns were restored in the compound mutants, indicating robust neurogenesis. Retinal tissue in I is bounded by the dashed lines. (M) The reduced eye size in the *R227W* mutant was partially rescued in the *R227W*; *Mitf^mi/+^* mutant at E12.5. (N) The expression of TUBB3 was detected in the *R227W*; *Mitf^mi/+^* retina at E13.5. (O) *Mitf* and *Otx1* transcript levels were much lower in the *R227W*; *Mitf^mi/+^* retina (black bars) compared to the *R227W* mutant (white bars). (P) pan-*Mitf* transcript level in *orJ; Mitf^mi/+^* retina was not lower than that in *orJ* retina. * P≤0.05; *** P≤0.001 Scale bars: 100 µm (A–F); 1 mm (insets); 50 µm (G–L); 0.5 mm (M); 100 µm (N).

To better understand the role of *Mitf* in microphthalmia and the retinal defects caused by the *Vsx2* mutations, we generated compound mutants homozygous for the *Vsx2* alleles and heterozygous for the *Mitf ^mi^* allele (*mi/+*). *mi* is a dominant negative allele and encodes a 3 bp deletion resulting in the loss of an arginine in the basic domain, which disrupts DNA binding in all Mitf isoforms [66], [67]. Early eye development proceeds relatively well in *mi/+* mice [68] and *orJ; mi/+* mice showed substantial improvements in eye size, tissue histology, and retinal neurogenesis by birth (Figure 7A, 7D, 7G, 7J). As expected, the degree of improvement of eye development in *R200Q; mi/+* was similar to *orJ; mi/+* (Figure 7B, 7E, 7H, 7K), but interestingly, eye size and retinal development in *R227W; mi/+* mice also improved to a level comparable with the other mutants (Figure 7C, 7F, 7I, 7L). Positive effects were observed early in retinal development as evidenced by increased eye size, circumference, and restored neurogenesis (Figure 7M, 7N). These findings were surprising because of the more profound consequences of the *R227W* mutation on eye development and the elevated levels of *Mitf* and *Otx1*. Consistent with the high degree of phenotypic rescue, pan-*Mitf* and *Otx1* expression levels were significantly reduced (Figure 7O). Importantly, pan-*Mitf* levels decreased disproportionately relative to the allele dosage. This was correlated with large reductions in all isoforms except *B-Mitf* (Table 1), which is already expressed at low levels [39]. This suggested that the enhanced *Mitf* expression in the *R227W* retina is due to positive feedback. This was unique to the *R227W* retina because *Mitf* levels were not decreased in the *orJ; mi/+* retina (Figure 7P). Our observations thus far reveal the essential and complex role of Mitf misregulation in causing microphthalmia and in interfering with retinal development in the Vsx2 mutant backgrounds.

**Table 1:** Relative retinal mRNA levels in *R227W* and compound mutants at E12.5.

	*R227W*	*R227W; Mitf^mi/+^*	*R227W, p27^+/−^*
**pan-** ***Mitf***	1.00±0.12	0.15±0.08***	0.52±0.05** ^; ^ #
***D-Mitf***	1.00±0.07	0.09±0.04**	0.27±0.05*** ^; ^ #
***A-Mitf***	1.00±0.12	0.14±0.06**	1.69±0.29##
***H-Mitf***	1.00±0.11	0.07±0.03***	0.78±0.23#
***B-Mitf***	1.00±0.09	0.80±0.40	0.92±0.20
***J-Mitf***	1.00±0.20	0.19±0.06**	1.12±0.29##
***Otx1***	1.00±0.11	0.37±0.13*	0.45±0.04**

* P≤0.05;

** P≤0.01;

*** P≤0.001 (compared to *R227W*).

# P≤0.05;

## P≤0.01 (compared to *R227W; Mitf ^mi/+^*).

### Genetic reduction of *p27^Kip1^* enhances eye size and restores neurogenesis in the *R227W* retina

**Figure 8:**
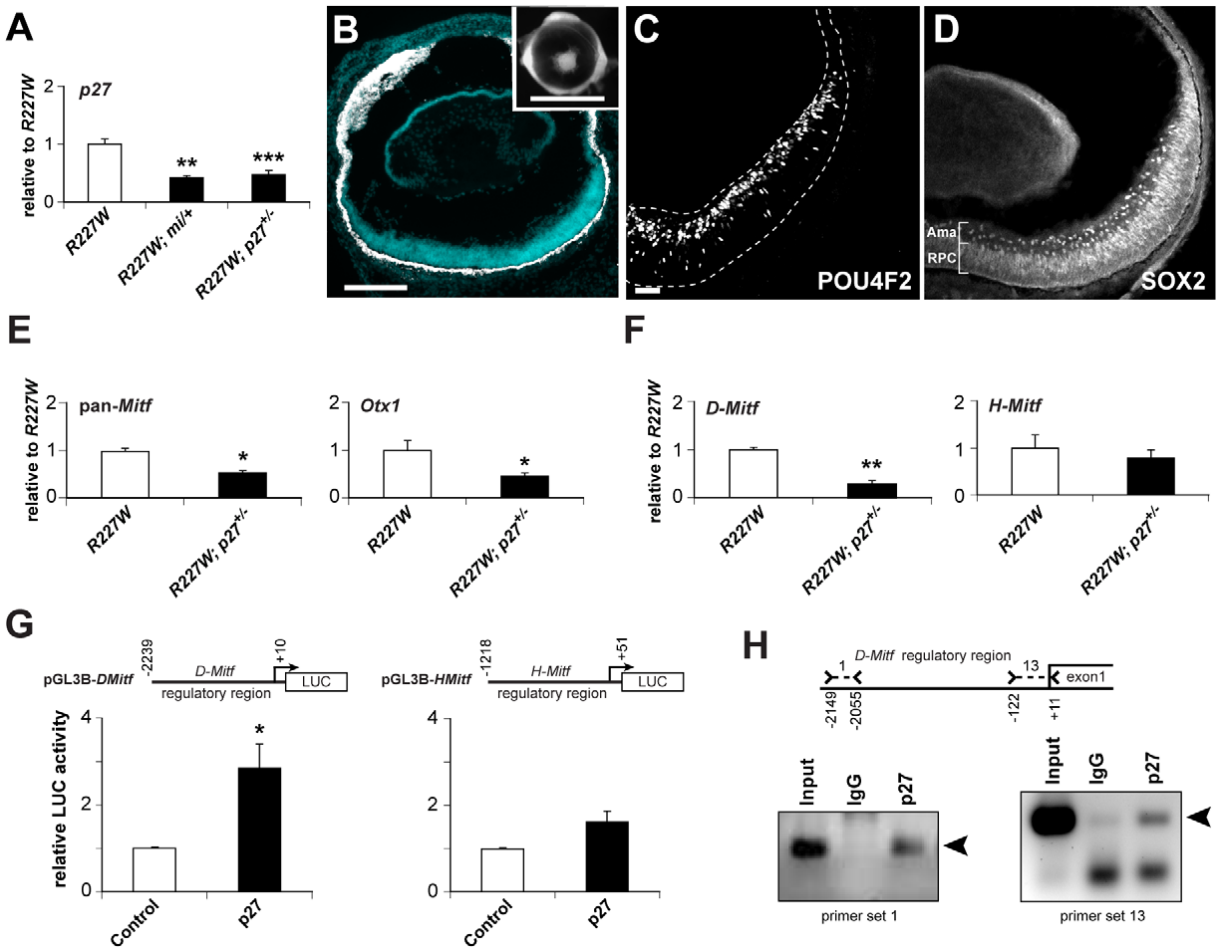
p27Kip1 is part of a gene regulatory network promoting pigmentation in the *R227W* retina. (A) *p27* mRNA was reduced by approximately half in *R227W*; *Mitf^mi/+^* and *R227W*; *p27^+/−^* compound mutant retinas compared to *R227W* at E12.5. (B–D) Phenotypes of *R227W*, *p27Kip1^+/−^* compound mutant eyes at P0. (B) Retinal tissue (blue) was restored along with a concomitant loss of pigmentation (white) in the retina. The peripheral retina was not rescued to the same extent as the central region. Eye size was also enhanced in the compound mutant (inset). POU4F2 (C) and the amacrine cell marker SOX2 (D) were expressed in the compound mutant. SOX2 is also expressed in RPCs. (E) Expression of *Mitf* and *Otx1* mRNAs were reduced by approximately half in compound mutant retinas (black bars) compared to *R227W* (white bars) at E12.5. (F) The expression level of *D-Mitf* mRNA was reduced in compound mutant retinas, whereas *H-Mitf* mRNA levels were were not significantly different. (G) p27 overexpression in HEK293 cells increased luciferase activity from pGL3B-*DMitf*, but to a much lesser extent from pGL3B-*HMitf*. (H) ChIP assays of E12.5 *R227W* retinal lysates probed with p27 antibody. ChIP panel on left shows products obtained with primer set 1; panel on right shows products obtained with primer set 13. Sequence-verified products denoted by arrowheads. * P≤0.05; ** P≤0.01; *** P≤0.001 Scale bars: 100 µm (B); 1 mm (inset); 50 µm(C,D).

We previously showed that genetic inactivation of the cyclin-dependent kinase inhibitor p27^Kip1^ (p27; MGI symbol: *Cdkn1b*) significantly enhances eye size and retinal development in *orJ* mice [50]. Although we didn't examine the effects of p27 removal on Mitf expression or retinal pigmentation in that study, we have yet to observe pigmentation in regions where retinal histogenesis was restored. This was also true for *R200Q; p27* double mutants (data not shown). To determine if p27 also contributed to the alterations in eye development and the pigmentation potential of *R227W* RPCs, we generated *R227W; p27* compound mutants. We analyzed retinas from *R227W* homozygous, *p27* heterozygous (*R227W; p27^+/−^*) mice because they expressed p27 at a level similar to that in *R227W; mi/+* retinas (Figure 8A). Interestingly, partial reduction in p27 was sufficient to inhibit pigmentation and promote retinal histogenesis (Figure 8B–8D). As in the *orJ;p27^−/−^* retina, the peripheral regions of the *R227W; p27^+/−^* retina was not rescued to the same extent as the central region. These data revealed that p27 was a key factor in promoting the *Vsx2* mutant phenotypes regardless of allele.

### Vsx2^[R227W]^, p27, H-Mitf, D-Mitf, and Otx1 form a transcriptional positive feedback loop

As with the *R227W; mi/+* retina, pan-*Mitf* and *Otx1* mRNA and protein expression levels dropped in the *R227W; p27^+/−^* retina (Figure 8E; Figure S6A, S6B; data not shown). There were notable differences, however, between the two rescue models (Table 1). In the *R227W; p27^+/−^* retina, the pan-*Mitf* level was intermediate to the *R227W* and *R227W; mi/+* retinas. Among the *Mitf* isoforms, only *D* was reduced in the *R227W; p27^+/−^* retina (Figure 8F; Table 1), whereas all *Mitf* isoforms (except *B*) dropped well below half in the *R227W; mi/+* retina (Table 1). Considering that *H-Mitf* was the isoform primarily responsible for the elevated increase in the pan-*Mitf* level in the *R227W* mutant over *orJ* (and *R200Q*), these data show that *H-Mitf* was not sufficient to drive the *R227W* phenotype when *D-Mitf* and *p27* levels dropped down. These data are consistent with a genetic pathway in which D-Mitf is downstream of p27, and p27 is downstream of H-Mitf .

Since *D-Mitf* mRNA expression decreased when one allele of *p27* was inactivated in the *R227W* retina, we asked whether this regulation could be direct. First, we overexpressed p27 with our *D-Mitf* luciferase reporter (pGL3B-*DMitf*) in HEK293 cells. In contrast to the repression observed with VSX2 and VSX2^[R227W]^ (Figure 1D), p27 enhanced reporter activity (Figure 8G, left graph). That this reflects a specific interaction is supported by our observation that p27 did not significantly alter reporter activity when Luciferase was expressed under the control of ∼1.2 kb of the *H-Mitf* promoter region (pGL3B-*HMitf*; Figure 8G, right graph). Second, we performed ChIP assays with an antibody against p27 and chromatin lysates isolated from E12.5 *R227W* retinas using 13 primer sets that covered ∼2.2 kb of 5′-intergenic sequence. PCR enrichment was observed with primer set 1 and primer set 13 (Figure 8H). These data suggest p27 acts directly to regulate *D-Mitf* transcription in *R227W* RPCs.

**Figure 9:**
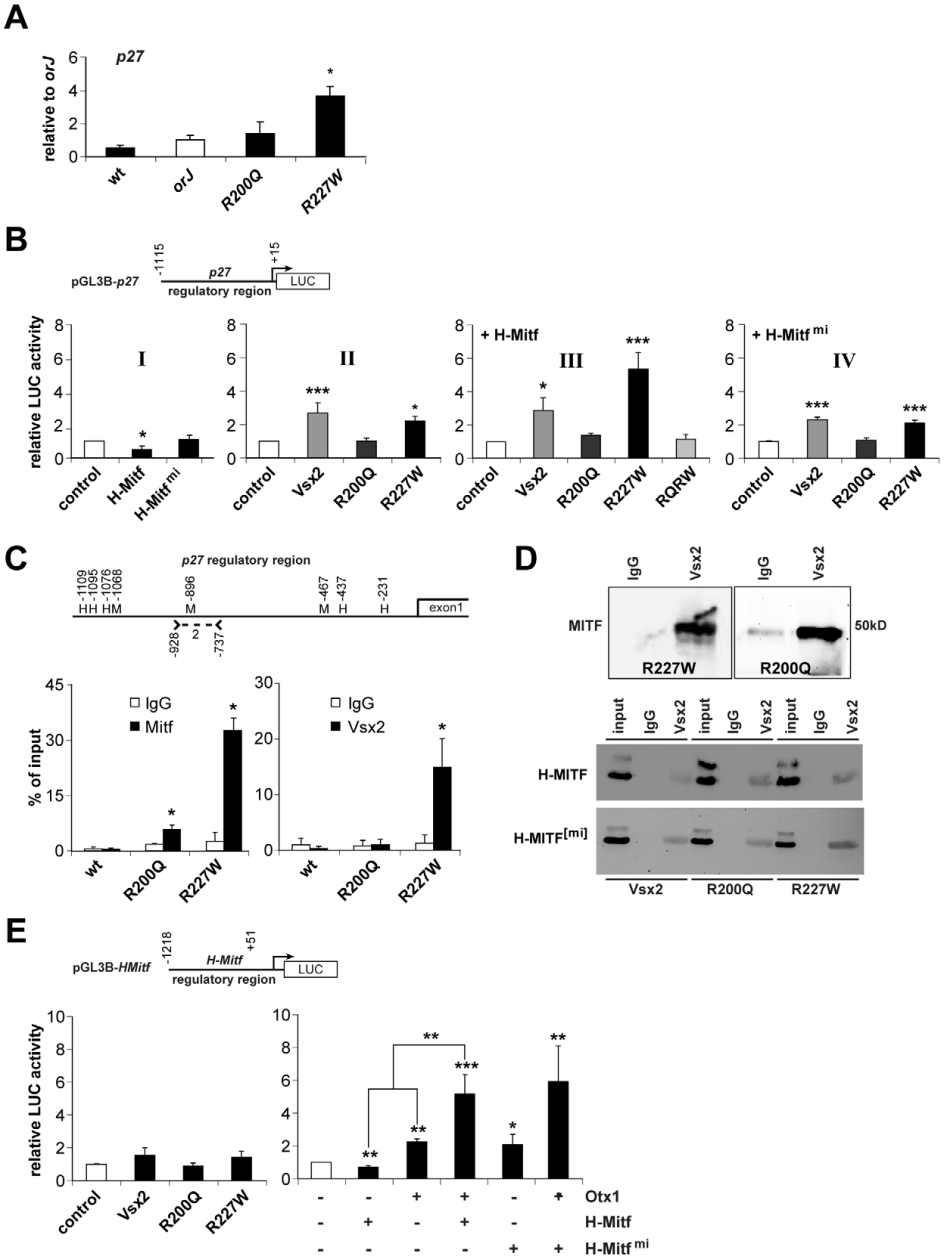
Molecular interactions between the *VSX2* variants and components of the pigmentation circuitry. (A) *p27* mRNA expression in E12.5 retinas of the indicated genotypes as determined by qRT-PCR. Samples were normalized to *orJ*. Only the *R227W* retina was significantly different. (B) Luciferase activities from HEK293 cells transfected with the indicated expression vectors (*x-*axes) and ∼1.1 kb of the *p27* promoter region (pGL3B-*p27*). Graph I: H-MITF repressed reporter activity in a DNA binding-dependent manner. Graph II: VSX2 and VSX2^[R227W]^ enhanced reporter activity. Graph III: H-MITF combined with VSX2^[R227W]^ elicited a specific and synergistic increase in reporter activity that depended on DNA binding as revealed by the abrogated activity of the VSX2^[R200Q, R227W]^ double mutant (RQRW). Graph IV: Expression of the *mi* version of H-MITF had no effect on reporter activity resulting from VSX2 or its variants. (C) Schematic of *p27* 5′-intergenic region (∼1.1 kb). Positions of putative Mitf binding sites (M) and homeodomain core sequences (H) are shown. Positions are relative to *p27* transcriptional start site. Position of primers that constitute *p27* primer set 2 (Table S1) is also shown. Graphs show quantification of ChIP-qPCR assays using MITF or VSX2 antibodies reacted with E12.5 lysates from wild-type, *R200Q* and *R227W* retinas. MITF binding was detected in *R200Q* and *R227W* lysates. VSX2 binding was detected in *R227W* lysate. (D) Co-IPs of E12.5 *R227W* and *R200Q* retinal protein lysates with a negative control sheep IgG or VSX2 antibodies followed by western blot probed with MITF antibody (top panel). Co-IPs of HEK293 cells transfected with VSX2 or its variants (listed below images) plus H-MITF (middle panel) or its *mi* variant (bottom panel). IPs were performed with sheep IgG or VSX2 antibodies followed by western blot probed with MITF antibody. *input* refers to the 20% of whole protein lysate used for co-IP. (E) Luciferase assays in HEK293 cells transfected with the indicated expression vectors (*x-*axes) and the pGL3B-*HMitf*. Left graph: effects of VSX2 and its variants on reporter activity were not statistically significant. Right graph: H-MITF repressed reporter activity, whereas OTX1 enhanced reporter activity. Reporter activity in cells co-expressing of OTX1 and H-MITF was significantly higher than the sum of the factors expressed individually (** associated with lines over bars). H-MITF^[mi]^ enhanced reporter activity, but reporter activity in cells co-expressing OTX1 and H- MITF^[mi]^ was not significantly different than the sum of the two factors expressed individually. * P≤0.05; ** P≤0.01; *** P≤0.001.

Since p27 is an important factor in promoting both the *orJ* and *R227W* phenotypes and yet the phenotypes differ in severity, we asked whether p27 is regulated differently in the two mutants. *p27* transcript levels were equivalent in wild-type and *orJ* retinas at P0 [50] and its expression was not significantly different between wild-type, *orJ*, or *R200Q* at E12.5 (Figure 9A). In contrast, *p27* mRNA and protein expression was higher in the *R227W* retina (Figure 9A; Figure S6D-S6G), with the highest expression in the peripheral regions, similar to MITF and OTX. These observations correlate p27 expression level with the phenotypic severity of the *R227W* mutant and indicate a novel mode of p27 regulation.

p27 is a candidate target of transcriptional activation by Mitf in chick RPE [69]. As our data suggested that p27 is downstream of H-Mitf, we tested whether H-Mitf enhances reporter activity using ∼1.1 kb of the *p27* promoter region (pGL3B-*p27*; Figure 9B). This was not the case, however, as H-MITF repressed reporter activity in a DNA binding-dependent manner (Figure 9B, graph I). We also tested VSX2 and its variants and found that both VSX2 and VSX2^[R227W]^ enhanced reporter activity whereas VSX2^[R200Q]^ had no effect (Figure 9B, graph II). Interestingly, when H-MITF was expressed with VSX2^[R227W]^, reporter activity increased further and beyond that observed for VSX2 (Figure 9B, graph III). This appeared to depend on DNA binding by VSX2^[R227W]^ because the enhancement was eliminated in a mutant protein containing both VSX2^[R200Q]^ and VSX2^[R227W]^ (RQRW; Figure 9B, graph III). The enhancement also depended on H-MITF DNA binding by since reporter activity was reduced when VSX2^[R227W]^ was co-expressed with H-MITF^[mi]^ (Figure 9B, graph IV).

The dependence of the enhanced reporter activity on DNA binding suggested that these proteins could bind in the p27 promoter region. To test this, we searched 1.1 kb of the p27 5′-intergenic region directly upstream of the transcriptional start site for Mitf and Vsx2 binding sites. Three Mitf consensus sequences (CANNTG) and five homeodomain core sequences (TAAT) were identified (Figure 9C). However, none of the homeodomain core sequences, when extended, matched the VSX consensus sequence (PyTAATTPuPu; Py, pyrimidine; Pu, Purine). We then performed a ChIP scan using 6 primer pairs that cover this intergenic region and identified one primer pair (primer set 2) that showed enrichment with the VSX2 and MITF antibodies in E12.5 *R227W* retinal chromatin lysates (data not shown). One of the putative Mitf binding sites was contained within the region amplified by primer set 2 (Figure 9C). Next, we performed ChIP-qPCR to determine the relative occupancies of VSX2 and MITF in this region in E12.5 wild-type, *R200Q*, and *R227W* retinal cells. As expected, MITF occupancy was not detected in wild-type but was detected in *R200Q* and *R227W* chromatin (Figure 9C, left graph). Importantly, the relative enrichment in MITF occupancy was higher in *R227W* chromatin and this was coincident with VSX2 occupancy, which was found only in *R227W* chromatin (Figure 9C, right graph).

The lack of a candidate VSX2 binding site or homeodomain core sequence in the ChIP-enriched region suggested that the interaction of chromatin and the VSX2^[R227W]^ protein could be indirect and mediated by an interaction with MITF. The switch in MITF activity from repressing to enhancing reporter activity in the presence of VSX2^[R227W]^ also suggested that an interaction between the two proteins was influencing *p27* transcriptional regulation. Co-immunoprecipitation (co-IP) of E12.5 *R227W* retinal lysates using the VSX2 antibody for IP and the MITF antibody for western blot revealed that this was indeed the case (Figure 9D, top left panel). Perhaps more surprising, this interaction was not unique to VSX2^[R227W]^ since VSX2^[R200Q]^ also co-immunoprecipitated with MITF in *R200Q* retinal lysates (Figure 9D, top right panel). Co-IP reactions were negative in the *orJ* retina, consistent with the lack of Vsx2 protein in this mutant (data not shown). Furthermore, all three VSX2 variants co-immunoprecipitated with H-MITF in transfected HEK293 cells and these interactions were not disrupted by the *mi* mutation (Figure 9D, bottom panels), which suggests that these interactions were intact in the *mi* and *Vsx2* compound mutants. The sum of these data suggests that the novel regulation of p27 expression in the *R227W* retina is dependent on the unique convergence of weak DNA binding by VSX2^[R227W]^, the normal DNA binding activity by MITF, and the interaction between the two proteins.

While we now have explanations for the elevated levels of *p27* and *D-Mitf* in the *R227W* mutant retina, how the *H-Mitf* level increased was still not clear. Although VSX2 is associated with chromatin in the *H-Mitf* regulatory region [39], reporter activity in HEK293 cells transfected with pGL3B-*HMitf* was not different between cells expressing VSX2 or its variants (Figure 9E, left graph). H-MITF was also not sufficient to enhance reporter activity, but rather caused moderate repression (Figure 9E, right graph). In contrast, OTX1 increased reporter activity and this was further enhanced by the presence of H-MITF (Figure 9E, right graph). This interaction was synergistic because the activity of OTX1 and H-MITF expressed together was greater than the sum of the activities of the factors expressed individually, and the P-value (0.004) from a two-way ANOVA supports this conclusion. Unexpectedly, the relative luciferase activity in cells co-expressing OTX1 and the H-MITF^[mi]^ was similar to the co-expression of OTX1 and wild-type H-MITF (Figure 9E, right graph). In this case, however, the effect was not synergistic (P = 0.09, two-way ANOVA), but rather reflected an additive effect since H-MITF^[mi]^ expressed alone also enhanced luciferase activity over the control (Figure 9E, right graph). These observations indicated that the synergistic effect of Otx1 and H-Mitf on the H-Mitf promoter was dependent on DNA binding by H-Mitf. In sum, the uniquely elevated level of H-Mitf in the *R227W* mutant was not likely due to the direct action of the VSX2^[R227W]^ protein at the *H-Mitf* promoter, but rather to the collaboration of Otx1 and H-Mitf, and possibly other Mitf isoforms.

## Discussion

We show that the homeodomain and CVC mutant proteins VSX2^[R200Q]^ and VSX2^[R227W]^ have diminished DNA binding capacities but retain other functions important for transcriptional activity such as nuclear localization and the ability to regulate transcription when fused to a heterologous DNA binding domain. As in humans, both mutations cause severe non-syndromic bilateral microphthalmia in mice thus confirming their pathogenicity. The recessive nature of these alleles in both species suggests that the alterations in the functional properties of VSX2 caused by each mutation are conserved. Our data argue that the homeodomain is dependent on the CVC domain for high affinity DNA binding highlighting the importance of non-homeodomain residues in mediating canonical homeodomain function. Furthermore, our data suggest that high affinity DNA binding by VSX2 is required to suppress the activation of transcriptional circuits that interfere with the retinal gene expression program in RPCs.

### Homeodomain-dependent DNA binding is required for VSX2 function

Two ways in which a mutation can alter the DNA binding properties of a protein are by changing the binding site preference (gain of function) or by reducing overall DNA binding affinity (loss of function). Our data support the contention that the VSX2^[R200Q]^ protein is DNA binding deficient: the VSX2^[R200Q]^ mutant protein failed to bind its preferred sites *in vitro* and *in vivo* and the *R200Q* allele is recessive. Important to note, however, is that the *Otx1* transcript level in the *R200Q* retina was not upregulated, as in the *orJ* and *R227W* retinas. On this basis, the *R200Q* allele behaves as a strong hypomorph since its phenotype at the cell and tissue levels overlaps with the null phenotype, but with at least one difference at the molecular level.

While the low level of *Otx1* also suggests that the VSX2^[R200Q]^ protein can still regulate some downstream factors, it is unlikely to accomplish this through direct DNA binding. R200 corresponds to residue 53 of the homeodomain and an arginine at this position (HD-R53) is among the most conserved residues in the homeodomain. HD-R53 forms hydrogen bonds with two phosphates in the DNA backbone and is not directly involved with sequence specificity. Rather it orients the recognition helix into the major groove such that other homeodomain residues can make base-specific contacts, thereby enabling stable and sequence-specific DNA binding [2], [3], [70], [71]. The glutamine substitution likely disrupted the ability of the mutant protein to productively dock in the major groove of the DNA in its binding site, thereby greatly reducing or eliminating its ability to directly regulate downstream target genes. Thus, the lack of *Otx1* upregulation in the *R200Q* retina could be the result of a more complex mechanism, such as by interfering with or altering the transcriptional regulation of an interacting partner such as Mitf.

### The CVC domain is required for high-affinity DNA binding by the homeodomain

Genetic data from humans, *C. elegans*, and now mouse confirm that the CVC domain is essential for the function of Vsx-class proteins. Our EMSA and ChIP data show that a single amino acid substitution in the CVC domain (R227W) weakened the DNA binding capacity of VSX2 to its preferred sites, indicating that optimal homeodomain function depends on the CVC domain. How the CVC domain assists the homeodomain is not clear. One possibility is that it recruits additional proteins required for DNA binding. Alternatively, the CVC domain may directly interact with the homeodomain to overcome a structural constraint on the homeodomain-DNA interaction, similar to that proposed for the C-terminal tail in PBX homeodomain proteins [12]. Structural limitations can be an intrinsic property of the homeodomain or the DNA binding site [14], but since the preferred binding sites of closely related homeodomain proteins such as Rx, Alx, and Arx overlap with the VSX proteins [10], and yet these proteins lack CVC domains, leads us to predict that any structural limitation will be endemic to VSX homeodomains.

### The *R227W* allele is a recessive neomorph that activates a cryptic transcriptional circuit through its interaction with Mitf

**Figure 10:**
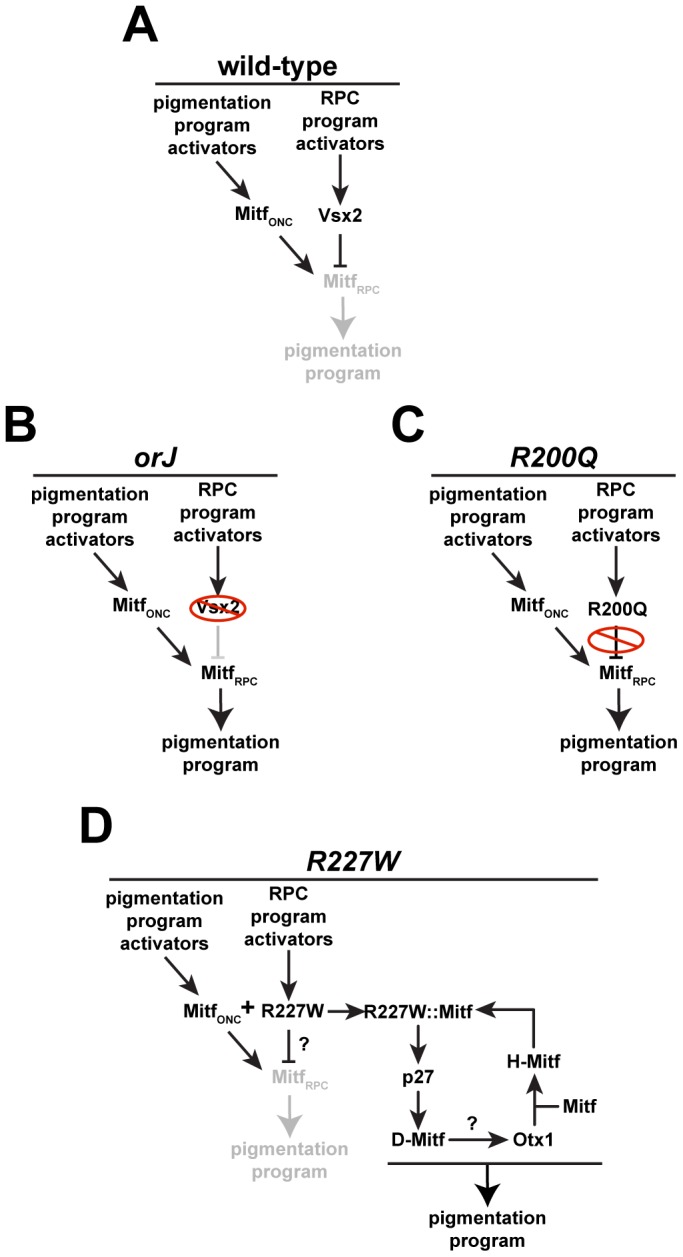
Regulation of pigmentation programs in wild-type and mutant RPCs. (A) During early eye development, Mitf is expressed in optic neuroepithelial cells (Mitf_ONC_) in response to upstream activators. Vsx2 expression is activated in the newly specified retinal domain by upstream activators, which leads to repression of Mitf in RPCs (Mitf_RPC_) and suppression of the pigmentation program. (B) In *orJ* mice, Mitf persists in RPCs because the VSX2 protein is absent, which increases the probability that pigmentation will occur. (C) In *R200Q* mice, VSX2^[R200Q]^ protein is present but unable to bind DNA, allowing Mitf to persist in RPCs, increasing the probability of pigmentation. (D) In *R227W* mice, VSX2^[R227W]^ protein is present and may still suppress the pathway that leads to pigmentation in *orJ* and *R200Q* RPCs, but its interaction with Mitf combined with its weak DNA binding activity engages a novel positive feedback loop that activates a robust pigmentation program. Our genetic data place Otx1 downstream of p27 and D-Mitf, but the mechanism causing its elevated expression is not clear.

Identifying the mechanisms underlying the mutant phenotypes revealed how *R227W* could surpass *orJ* and *R200Q* in severity (Figure 10). During normal eye development, Mitf expression is activated in optic neuroepithelial cells (Mitf_ONC_) of the optic vesicle, presumably by signals that promote RPE specification and the pigmentation program [72]. This is followed by activation of Vsx2 in the presumptive retinal domain, which leads to repression of Mitf activity in RPCs (Mitf_RPC_), effectively blocking the execution of the pigmentation program in these cells (Figure 10A). In *orJ* and *R200Q* mice, the ability to repress Mitf in RPCs is lost, leading to maintained Mitf activity and an increased probability that a pigmentation program will be activated. The primary differences are that in *orJ* mice, VSX2 protein is absent (Figure 10B) and in *R200Q* mice, VSX2^[R200Q]^ protein is present but unable to bind DNA (Figure 10C). Like the *orJ* and *R200Q* mutants, Mitf expression also persists in early RPCs of the *R227W* mutant. Our *in vitro* reporter data indicates that the VSX2^[R227W]^ protein can still repress *D-Mitf*, but the *in vivo* ChIP data suggests that its weak DNA binding may reduce or abrogate its repressive effects on the endogenous *D-Mitf* promoter. Ultimately, our data suggests the interaction between the VSX2^[R227W]^ and Mitf proteins activates a positive feedback loop that significantly elevates the expression of p27, Otx1 and Mitf, leading to a robust pigmentation program and a blocked retinal program in *R227W* RPCs (Figure 10D). Our genetic data also suggests that p27 is a novel target specific to the R227W:MITF complex, even though Mitf is proposed to be a direct regulator of p27 expression in chick RPE [69].

Mechanistically, manifestation of the *R227W* phenotype likely occurs in two steps; the weakened DNA binding activity first acts as a partial loss-of-function that is sufficient to allow persistent Mitf expression. This is followed by the activation and maintenance of the feedback loop, which depends on the protein interaction between the VSX2^[R227W]^ and MITF proteins. Since the MITF interaction is shared among the three VSX2 variants, it by definition is not a gain-of-function activity, which suggests there must be another activity unique to the VSX2^[R227W]^ variant. This novel activity could be the result of the unique combination of the weak DNA binding caused by the *R227W* mutation and the interaction with MITF, ultimately leading to a change in transcriptional regulation of downstream targets, novel or otherwise. The VSX2^[R227W]^ protein may have acquired novel off-target binding properties allowing it regulate a suite of genes not normally regulated by Vsx2. While possible, this is unlikely on its own to explain the phenotype because acquisition of new binding site preferences is typically revealed as a dominant or semi-dominant gain of function phenotype, which was not observed. In addition to its effects on DNA binding, the *R227W* mutation may have induced a conformational change in the VSX2:MITF complex that alters how the complex interacts with DNA and regulates transcription. It is also possible that another interacting partner is involved and is present only in the *R227W* mutant. Distinguishing between these possiblities requires further genetic and molecular analyses.

Since the positive feedback loop is not found in the *orJ* or *R200Q* mutants, it is by definition a novel mechanism. Combined with the genetic properties then, the *R227W* allele best fits the classification of a recessive neomorph. Although predicted to exist over 80 years ago by H.J. Muller [73], reports of recessive neomorphic alleles are difficult to find. Recently, strong evidence for another atypical allele, the *recessive antimorph*, was reported for the CXC domain-containing gene *TSO1* in *Arabidopsis* [74]. It is likely that more recessive neomorphs and antimorphs will be identified as targeted alleles are generated in genetic models that mimic disease-linked missense mutations found in natural populations. Their identification will require direct comparison to a corresponding null allele and may be more easily identified at loci that are highly susceptible to missense mutations and show a wide range of phenotypes.

### A novel role for p27 as a regulator of transcription

In identifying the molecular mechanism driving the *R227W* phenotype, we uncovered evidence that p27 regulates transcription, specifically of the *D-Mitf* isoform. This was unexpected because *D-Mitf* levels are not dependent on p27 in the *orJ* retina (data not shown), indicating that this functional link is context-dependent. Consistent with this, partial genetic reduction in p27 expression in the *R227W* retina reduced *D-Mitf* expression, which suggests that the elevated expression of p27 unmasked its transcriptional activity. It is not yet clear if p27 regulates transcription in the wild-type retina or if this is a common feature of p27 function. There are hints, however, that support these possibilities. p27 enhances reporter activity driven by *Myelin Basic Protein* promoter in an Sp1-dependent manner [75], [76]. p27 also binds to and stabilizes Neurogenin 2, a pro-neurogenic bHLH transcription factor [77]. In the retina, Cyclin D1 was recently shown to bind to chromatin and regulate genes such as Notch1 [78]. As Cyclin D1 and p27 interact genetically and biochemically [79], [80], it is conceivable that misregulation of transcriptional targets could be a causative factor in the alterations in retinal development found in Cyclin D1 and p27 knockout mice [61], [81]–[84].

### Vsx2 acts as a gatekeeper allowing the retinal progenitor program to proceed without interference from other gene expression programs

The neuroepithelial-derived components of the eye field develop through a process of progressive specification from the anterior neuroectoderm and Vsx2 expression is arguably the strongest indicator of retinal domain specification [72]. To date, it is the only transcription factor expressed exclusively and comprehensively in the retinal domain and its onset of expression immediately precedes the formation of the optic cup, which coincides with the earliest morphological changes that distinguish RPCs from the remainder of the optic neuroepithelium [17], [54]. Since Vsx2 expression initiates well after eye field specification, it stands to reason that activation of the RPC program is an actively promoted process. Consistent with this, eye field specification occurs and optic vesicles form in the *Lhx2* mutant, but Vsx2 is not expressed and the RPC program fails to initiate [54]. Rather, the more likely fate following eye field specification is pigmented epithelium. Mitf is expressed throughout the optic neuroepithelium prior to Vsx2 expression and in mice in which FGF or BMP7 signaling is disrupted, Mitf expression persists in the presumptive retinal domain, which correlates with the failure to express Vsx2 and a high probability of pigmentation where the retina would normally form [72], [85], [86].

Despite these findings, Vsx2 does not act as a master regulator to specify or activate the RPC program because the retinal domain and optic cup still form in all three Vsx2 mutants. Retinal development, although compromised, still occurs in the absence of VSX2 protein (*orJ* mouse) and this is not due to genetic compensation or functional redundancy by *Vsx1* [32]. Importantly, the *Vsx2* gene is still expressed in the *Vsx2* mutants, indicating that initiation of the RPC program is independent of Vsx2 function, or in the case of the *R227W* mutant, despite the neomorphic behavior caused by the mutant protein. Thus, a primary role of Vsx2 is not to initiate specification, but rather to allow the RPC program to proceed without interference from competing gene expression programs. A failure to block these programs may not convert RPCs to another well-defined fate *per se*, but they hinder the RPC program and lead to, in some contexts, to aberrant differentiation as revealed by the pigmentation phenotype.

**Figure 11:**
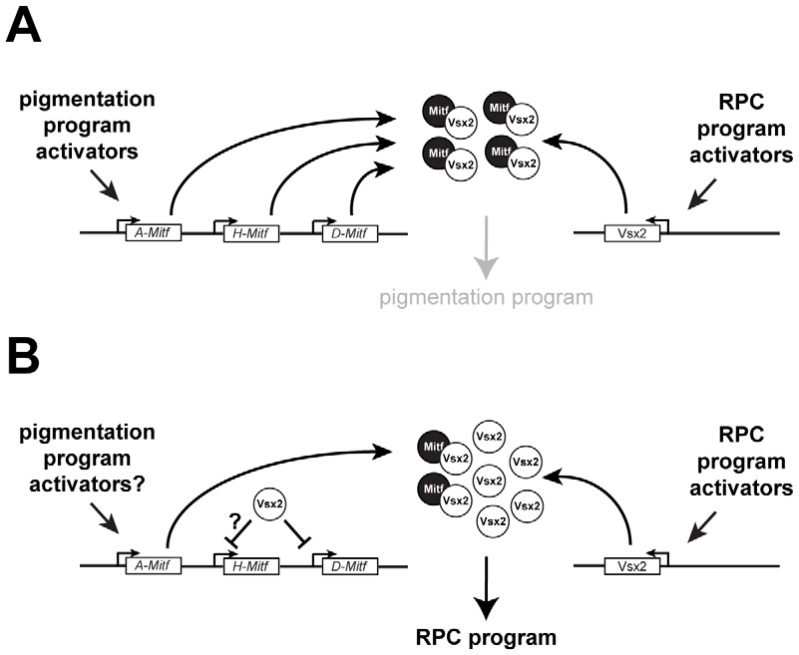
Models of Mitf regulation in RPCs during normal retinal development. (A) In response to RPC program activators, Vsx2 expression is initiated and newly produced protein interacts with preexisting MITF protein, preventing access to targets required for the pigmentation program. (B) Once VSX2 protein expression is established, it regulates Mitf activity by directly repressing *Mitf* transcription of isoforms such as *D-Mitf* and binds to MITF proteins produced from promoters that Vsx2 does not efficiently repress such as *A-Mitf* and possibly *H-Mitf*.

Antagonizing Mitf activity through transcriptional repression is a key function for Vsx2 in this process. While a likely mechanism for D-Mitf regulation (this study; [39]) other Mitf isoforms are also misexpressed in the *Vsx2* mutant retinas and our data suggest that the H and A isoforms are not transcriptionally regulated by Vsx2. Supporting this last point is that A-Mitf is expressed in the wild-type retina during development [39]. Although A-Mitf may be expressed below a threshold needed to disrupt the RPC program, it is noteworthy that in vivo overexpression of Mitf failed to interfere with retinal development as long as Vsx2 was expressed [41]. This suggests that an additional Vsx2-dependent mechanism exists for antagonizing Mitf activity and we propose that it requires VSX2 binding to MITF, preventing MITF from regulating genes important for pigmentation. This mechanism could be important during the initial specification of the retinal domain, when Vsx2 has not had enough time to efficiently downregulate Mitf activity through transcriptional repression (Figure 11A), and during retinal histogenesis for Mitf isoforms that are not transcriptionally repressed by Vsx2 (Figure 11B). In effect, this enables Vsx2 to block Mitf from interfering with the execution of the RPC program by regulating its activity at two levels. Interestingly, the functional significance of the protein interaction between Vsx2 and Mitf may also depend on high affinity DNA binding by Vsx2, which was suggested by the behavior of the VSX2^[R200Q]^ and VSX2^[R227W]^ proteins; both interacted with MITF but were unable to prevent Mitf-dependent alterations in retinal development. In the case of VSX2^[R227W]^, the interaction likely contributed to the more severe Mitf-dependent effects.

In sum, this study provides an explanation for the stable association of the homeodomain and CVC domain in the VSX proteins through evolution. Our mutational analysis suggests that an essential function of the CVC domain is to assist the homeodomain in achieving high affinity binding to its preferred sites. Although the specific downstream targets of the VSX proteins may vary in vertebrates and invertebrates, the molecular requirement for the CVC domain in allowing high affinity DNA binding by VSX homeodomains was likely to have been established much earlier in animal evolution than the innovation of the vertebrate eye. In the case of Vsx2, the stability of the homeodomain-CVC domain arrangement may have provided a platform for Vsx2 or its archetype to acquire the ability to efficiently regulate a complex locus such as Mitf, a key step in mammalian eye organogenesis and retinal development. Finally, our study shows that the generation of a small, but targeted allelic series of mutations in mice has the potential to reveal insight into protein function and human disease etiology.

## Materials and Methods

### Ethics statement

Procedures involving mice were approved by the Institutional Animal Care and Use Committee at the University of Utah and conformed to the standards outlined in the ARVO Statement for the Use of Animals in Ophthalmic and Vision Research.

### Mice

*Vsx2^orJ^* (129/SvJ genetic background) and *Mitf^mi^* (B6C3Fe genetic background) mice were obtained from the Jackson Laboratories (Bar Harbor, ME). *p27* knockout mice (129/C57Bl6 hybrid genetic background) were provided by Drs. Matthew Fero and James Roberts (Fred Hutchinson Cancer Center, Seattle, WA). *Vsx2^R200Q^* chimeric mice were generated at inGenious Targeting Laboratory (Stony Brook, NY) and *Vsx2^R227W^* chimeric mice were generated at the University of Utah Gene Targeting and Transgenic Core Facility (See Text S1 and Figure S1 for details on gene targeting strategy). Germline transmission into the 129/SvJ genetic background was achieved by breeding chimeric mice with mice from the *orJ* strain. All alleles were identified by PCR genotyping (primers listed in Table S1). For timed matings, females were considered to be at 0.5 days of gestation (E0.5) at noon on the day a vaginal plug was detected.

### Proliferation assays

P0 retinal cells from wild-type or *orJ* mice were dissociated with trypsin and trituration and cultured in DMEM/F12 medium with 1% fetal bovine serum (FBS), growth supplements, and a penicillin/streptomycin mix (Invitrogen) [87]. This age was selected for these and other culture experiments in order to maximize the cell yield on the days the cultures were established. Cells were transfected one day after plating (cell confluence ∼80%) with Lipofectamine and Plus Reagent (Invitrogen). Plasmids (Table S2) were transfected in equal amounts (0.6 µg per well in a 24-well plate, 0.2 µg per well in a 96-well plate). BrdU (10 µg/ml) was added for the last 4.5 hours of the culture period, starting at 44 hr post-transfection. Cultures were fixed with 4% PFA and stained with GFP, VSX2 and BrdU antibodies (Table S3). The percentages of BrdU+ cells in the VSX2+ or GFP+ cell populations were determined. A minimum of three independent trials was performed. Data were graphed as mean±SE. Statistical comparisons were done with the Kruskal-Wallis test. For simplicity, only statistical comparisons of the test conditions (black bars) versus the control (white bar) are shown.

### Luciferase assays

Dissociated P0 retinal cells were plated onto poly-D-lysine and 15 µg/ml laminin in the same medium as described above. HEK293 cells were cultured in DMEM supplemented with 10% FBS, 100 U/ml penicillin, and 100 µg/ml streptomycin (Invitrogen). In both cases, cells were transfected one day after plating (cell confluence ∼80%) with Lipofectamine and Plus Reagent. Plasmids are listed in Table S2. To compare reporter activity across conditions, expression vectors were transfected at equimolar concentrations and unless noted, empty expression vectors served as controls. Transfection efficiency was monitored by co-transfection of a Renilla luciferase reporter (Table S2). Cell lysates were prepared 24 h after transfection. Firefly and Renilla luciferase activities were measured with a Dynex Technologies MRX Revelation microplate reader (Dynex Technologies, Denkendorf, Germany) using 100 µl d-luciferin reagent and 100 µl coelenterazine (Biotium, Hayward, CA). Relative luciferase activity was calculated by using the control condition luciferase activity as a normalization factor for each independent trial (performed in duplicate). A minimum of three independent trials was performed for each condition. Data were graphed as mean±SE. Statistical comparisons were done with Fisher's Least Significance Difference (LSD) test and P-value ranges are shown only for test conditions (black bars) versus control (white bar) with the following exceptions: unpaired t-test was used for comparisons of test conditions versus control in Figure 8G and Figure 9E (right graph), and two-way ANOVA was used to test for synergistic interactions between H-Mitf and Otx1, and H-Mitf^mi^ and Otx1 (Figure 9E, right graph).

### CAT assays

HEK293 were cultured as described for Luciferase assays. 5 µg of LexA-Vsx2, -R200Q, -R227W or an equimolar amount of LexA were co-transfected with 0.5 µg of Gal4-HSF1 and 0.5 µg of X4G2CAT in 60-mm dishes at ∼50% confluency with Lipofectamine and Plus reagent. CAT assays were performed 24 h post-transfection. The amount of lysate used for CAT analysis was determined by quantifications of western blots of Gal4-HSF1 in ImageJ (http://imagej.nih.gov/ij/). The CAT activity was counted in an LS 6000IC liquid scintillation system (Beckman, Fullerton, CA) 60 min after the addition of ^[3H]^acetyl-CoA (Sigma-Aldrich, St. Louis, MO). Relative CAT activity was calculated by using control condition CAT activity as a normalization factor for each independent trial. Each trial was performed in duplicate and three independent trials were performed. Data were graphed as mean±SE. Statistical comparisons were done with Fisher's Least Significance Difference (LSD) test and P-value ranges are shown only for test conditions (black bars) versus control (white bar).

### Explant cultures

Ocular tissues were dissected from E10.5 embryos in HBSS. To implant POM, a local separation of the retina and lens was made with a 32G needle and POM was placed into the separated region. Explants were grown in the culture medium described for dissociated retinal cells. At the end of the culture period (2 days), explants were processed for immuohistology or retinas were dissected free of other tissues and prepared for qRT-PCR.

### Immunohistology

Heads, eyes, or retinas were fixed with 4% PFA and cryopreserved [61]. Sections were cut at a thickness of 12 µm. Primary antibodies are listed in Table S3. Primary antibodies were followed with species-specific secondary antibodies conjugated to Alexa Fluor 488 (Invitrogen) or Alexa Fluor 568 (Molecular Probes). Nuclei were stained with 4, 6-diamidino-2-phenylindole (DAPI; Sigma-Aldrich).

### Visualization of pigmentation in combination with fluorescence-based images

Images of DAPI stained sections were captured with epi-fluorescence illumination followed by bright-field illumination (BF). BF images were adjusted in Photoshop (Adobe, San Jose, CA) to high contrast and pixel values were converted to their inverse using the *invert image* function. DAPI images were made transparent with the *screen layer* setting and the images were merged.

### Eye circumference measurements

Eye circumference was assessed in image J and graphed as mean±SE. Statistical comparisons were done with the Kruskal-Wallis test.

### Quantitative real-time RT–PCR (qRT–PCR)

Unless noted, retinal tissue was dissected from the surrounding RPE, POM, and lens following treatment with 40 µg/ml dispase (Sigma-Aldrich) in HBSS for 5 min at room temperature. Total RNA was isolated with the RNeasy Mini or Micro Kit according to the manufacturer's instructions (Qiagen, Valencia, CA). cDNAs were prepared with the Superscript RT III Kit (Invitrogen) using 20 ng of total RNA and purified with the PCR purification kit (Qiagen). cDNA corresponding to 2 ng of total template RNA was used for each PCR reaction.

PCR was done on an ABI 7300 Real-Time PCR System with Power SYBR green PCR Master Mix and using the Relative Standard Curve Method for detection and measurement (Applied Biosystems, Invitrogen). Optimization was performed with serial dilutions of cDNA prepared from *orJ* retina. *Gapdh* was used as the endogenous control mRNA for all samples. RNA preparations from *orJ* or *R227W* retinas were used as calibrator samples. PCR product specificity was confirmed by gel electrophoresis and sequencing. Primer sets are listed in Table S1.

For each trial, measurements were calculated as the expression level (per condition) normalized to the calibrator sample. The mean±SE of the independent trials for each condition was then normalized by the mean of the control group and graphed as “relative to [control]” ([control] = *orJ* or *R227W*). Each trial was performed in duplicate and a minimum of three independent trials were performed with the following exceptions: the expression levels of Otx1 and Otx2 in the wild-type retina were measured in two independent samples (Figure 5J). Statistical comparisons were done with the Kruskal-Wallis test and P-value ranges are shown only for test conditions (black bars) versus control (white bar) with the exception of Figure 6H, which shows P-value ranges for all comparisons.

### Electrophoretic mobility shift assays (EMSA)

Expression plasmids are listed in Table S2. Proteins were synthesized by in vitro translation (ivt) using the EcoPro T7 System (Novagen, Madison, WI) and relative yields were estimated by western blot analysis probed with anti-VSX2 and detected with SuperSignal West Dura Extended Duration Substrate (Thermo Scientific, Rockford, IL). [^32^]P end-labeled double stranded P3 oligonucleotide (Table S1) was used to assess DNA binding [32]. The signals for Western blot and EMSA were captured on BioMax Light Film (Kodak, Rochester, NY).

### Immunoprecipitations (IP)

Non-denatured native protein lysates from E12.5 retinas with lens or transfected HEK293 cells were prepared RIP buffer (Santa Cruz) on ice for 60 min. ∼40 retinas were pooled for each lysate preparation. Equivalent volumes of cell lysates (input) were incubated with VSX2 antibody or sheep IgG at 4°C for 1 hour or overnight followed by Protein-G agarose (Roche) for 1 hour. Immunoprecipitated proteins were analyzed by Western blot using Mitf antibody. Signals for Western blot were captured by chemiluminescence on a ChemiDoc XRS (Bio-Rad, Hercules, CA).

### Chromatin immunoprecipitations (ChIP)

ChIP assays were performed on native retinal lysates as previously described [32]. Retinas with lens were isolated from E12.5 wild-type, R200Q or R227W eyes, and retinas were isolated from P0 wild-type eyes and prepared for ChIP assay. Antibodies are listed in Table S3, primer sets in Table S1. Quantative real time PCR (qPCR) using the Relative Standard Curve Method was performed to assess the relative strengths of protein∶chromatin interactions in each genotype. Results were expressed as the percentage enrichment normalized to the equivalent starting material (input). Statistical comparisons were done with the Kruskal-Wallis test and P-value ranges are shown for test conditions (black bars) versus IgG controls (white bars) in each genotype.

## Supporting Information

Figure S1 Generation of *R200Q* and *R227W* mutant mice. (A) Sequence tracks of PCR products encompassing the *R200Q* or *R227W* mutations. Asterisks denote base substitutions. Template DNA was prepared from mouse-tail DNA (wild-type) and targeted ES clones (mutants). (B) Schematic representation of the targeting strategy. The *R200Q* and *R227W* mutations (asterisk) were introduced into exon 4 and the ACN cassette [46] was cloned into intron 3 of Vsx2. These elements were then placed into the p*TK1TK2* targeting vector [88]. After homologous recombination, *R200Q-ACN* and *R227W-ACN* chimeric mice were generated. The ACN cassette was deleted by Cre recombination in the male germline generating the *R200Q* and *R227W* alleles. Chimeric males were crossed with wild-type females to generate germline-transmitted *R200Q* and *R227W* heterozygous mice. B: BamHI, H: HindIII, N: NheI. (C) Southern blots of genomic DNA from electroporated ES cell clones containing homologously recombined *R200Q-ACN* and *R227W-ACN* insertions. BamHI digestion and 3′ flanking probe (shown in B) were used. The wild-type band is 6.7 kb and the correctly targeted band is 7.6 kb. (D) Genotyping of mutant mice. P1 and P2 primers shown in B (arrows) were used for PCR genotyping. As the mutant allele contains a 34 bp insertion, wild-type and mutant alleles give 258 bp and 292 bp bands, respectively.
(TIF)

Figure S2 Proliferation changes associated with Vsx2^[R200Q]^ and Vsx2^[R227W]^ overexpression. (A) Micrographs showing expression of enhanced Green Fluorescent Protein fused to a nuclear localization signal (nlsGFP), VSX2, VSX2^[R200Q]^, and VSX2^[R227W]^ in transfected P0 *orJ* retinal cells. (B) Phosphorylated histone H3 (pHH3) expression was reduced in the mutant retinas compared to wild-type. (C) Quantification of BrdU incorporation in P0 *orJ* retinal cells overexpressing nlsGFP (control), VSX2, VSX2^[R200Q]^, or VSX2^[R227W]^. (D) Quantification of BrdU incorporation in P0 wild-type retinal cells overexpressing nlsGFP (control), VSX2, VSX2^[R200Q]^, or VSX2^[R227W]^. Cells were transfected at 24 hr following initial plating and cultured for an additional 48.0 hr. BrdU was added for the last 4.5 hr of the culture period. Only wild-type VSX2 enhanced proliferation over the control. * P≤0.05; ** P≤0.01 Scale bars: 100 µm.
(TIF)

Figure S3 Marker expression in wild-type, *orJ*, *R200Q*, and *R227W* retinas at P0. (A–D) Neurofilament-M (NF-M) expression in inner retinal neurons (differentiated cell layer, DCL) and horizontal cells (horiz), which occupy the neuroblast layer (NBL), was present in all genotypes except *R227W*. Similar results were obtained for the retinal ganglion cell marker POU4F2 (E–H) and the proliferation/RPC marker PCNA (I–L). Dashed lines bound retinas. Scale bar: 100 µm.
(TIF)

Figure S4 Marker expression in wild-type and *R227W/+* retinas at P0. Expression patterns of TUBB3 (A,B), NF-M (C,D), and SOX2 (E,F) in wild-type and R227W/+ retinas were similar. Scale bars: 100 µm.
(TIF)

Figure S5 Invasion of POM into the vitreal chamber in *R227W* eyes. (A) PITX2^+^ cells filled the vitreal chamber in the E17.5 *R227W* eye. (B) The relative expression levels of *D-Mitf* and *J-Mitf* were not statistically different between control (−POM) and POM implanted whole retina and lens explant (POM imp) cultures (E10.5+2DIV).
(TIF)

Figure S6 Immunolocalization of MITF and p27 in selected genotypes at E12.5. MITF expression in *R227W* (A) and *R227W; p27^+/−^* (B) eyes. (C) No primary antibody control. p27 expression in wild-type (D), *orJ* (E), *R200Q* (F), and *R227W* (G) eyes. Antigen retrieval was used for p27 staining to reveal staining in neuroblast layers. Bright staining at inner surface of central retina of wild-type is newly generated postmitotic precursors. Bright staining at the periphery of the *R200Q* retina is also observed occasionally in *orJ* retina at this age. p27 is also abundantly expressed in lens and surrounding extraocular tissues.
(TIF)

Table S1 Oligonucleotides.
(DOC)

Table S2 Plasmids.
(DOC)

Table S3 Primary antibodies.
(DOC)

Text S1 Materials and methods for generation of *R200Q* and *R227W* knock-in mice.
(DOCX)
