# Supplementary figures and images for: Vsx2 Controls Eye Organogenesis and Retinal Progenitor Identity Via Homeodomain and Non-Homeodomain Residues Required for High Affinity DNA Binding
Source: PLoS Genet. 2012 Sep 20;8(9):e1002924. doi: 10.1371/journal.pgen.1002924 (PMC3447932; doi:10.1371/journal.pgen.1002924)

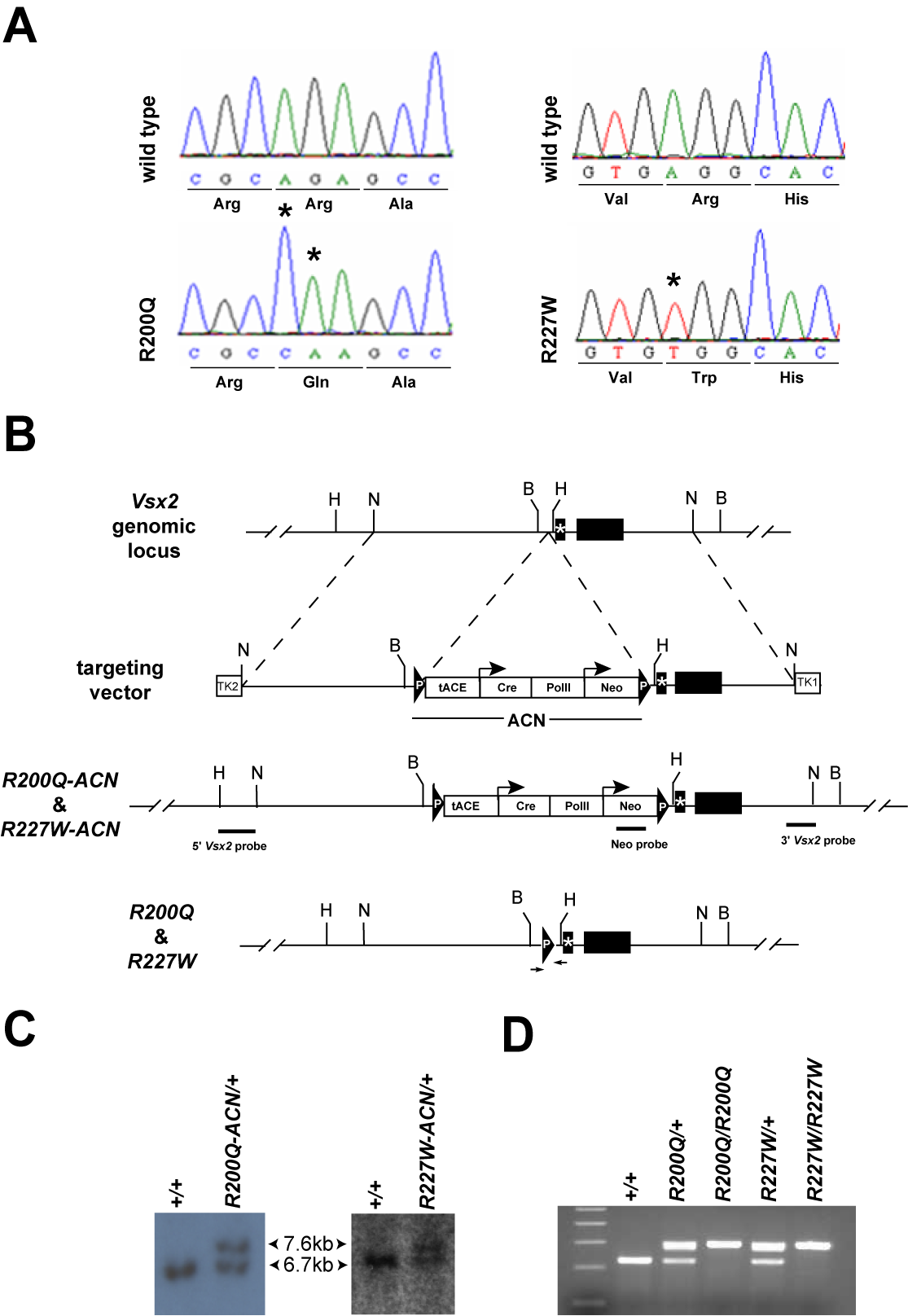

Supplement: Figure S1 — Generation of R200Q and R227W mutant mice. (A) Sequence tracks of PCR products encompassing the R200Q or R227W mutations. Asterisks denote base substitutions. Template DNA was prepared from mouse-tail DNA (wild-type) and targeted ES clones (mutants). (B) Schematic representation of the targeting strategy. The R200Q and R227W mutations (asterisk) were introduced into exon 4 and the ACN cassette [46] was cloned into intron 3 of Vsx2. These elements were then placed into the pTK1TK2 targeting vector [88]. After homologous recombination, R200Q-ACN and R227W-ACN chimeric mice were generated. The ACN cassette was deleted by Cre recombination in the male germline generating the R200Q and R227W alleles. Chimeric males were crossed with wild-type females to generate germline-transmitted R200Q and R227W heterozygous mice. B: BamHI, H: HindIII, N: NheI. (C) Southern blots of genomic DNA from electroporated ES cell clones containing homologously recombined R200Q-ACN and R227W-ACN insertions. BamHI digestion and 3′ flanking probe (shown in B) were used. The wild-type band is 6.7 kb and the correctly targeted band is 7.6 kb. (D) Genotyping of mutant mice. P1 and P2 primers shown in B (arrows) were used for PCR genotyping. As the mutant allele contains a 34 bp insertion, wild-type and mutant alleles give 258 bp and 292 bp bands, respectively. (TIF) [file pgen.1002924.s001.tif]

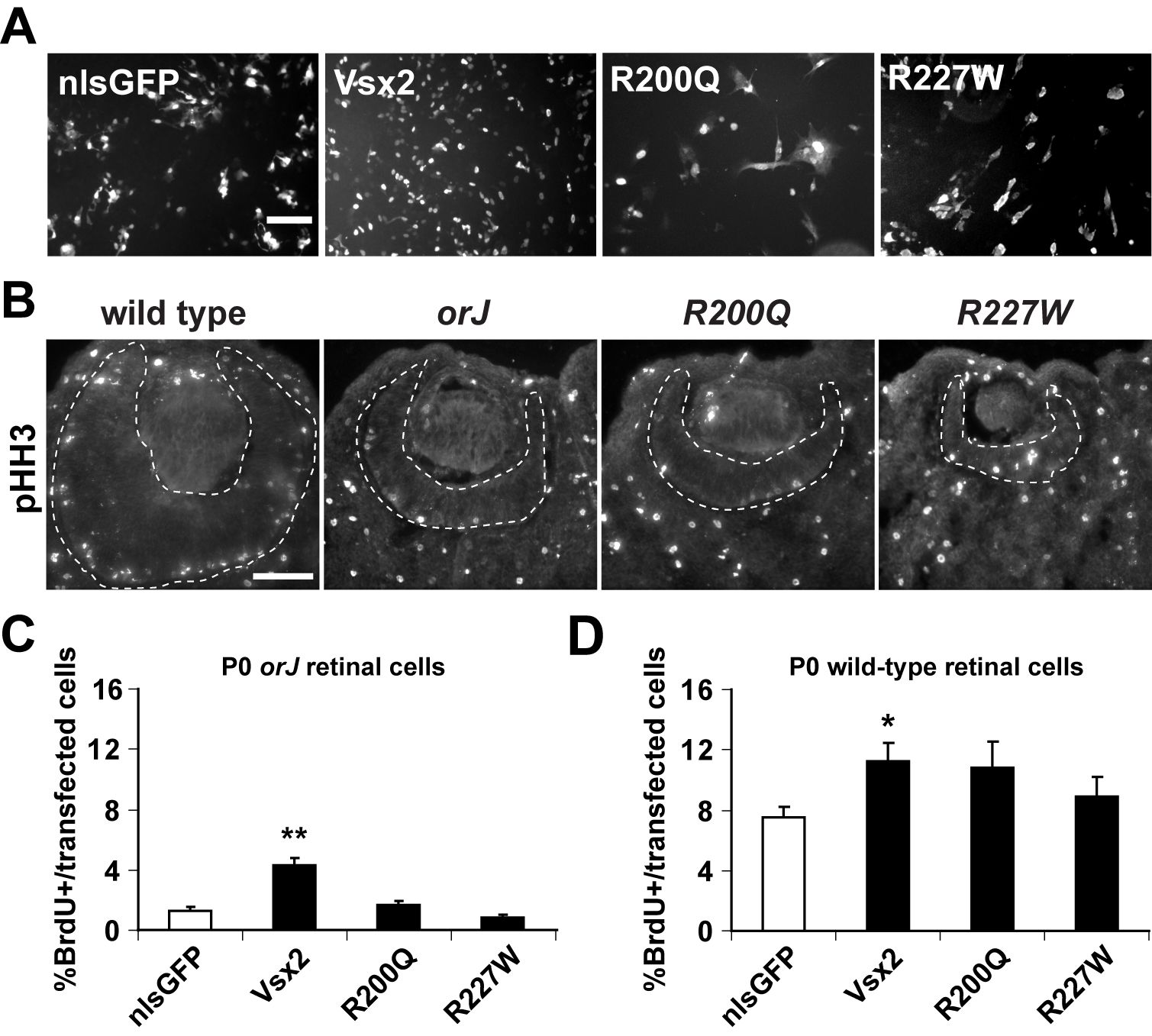

Supplement: Figure S2 — Proliferation changes associated with Vsx2[R200Q] and Vsx2[R227W] overexpression. (A) Micrographs showing expression of enhanced Green Fluorescent Protein fused to a nuclear localization signal (nlsGFP), VSX2, VSX2[R200Q], and VSX2[R227W] in transfected P0 orJ retinal cells. (B) Phosphorylated histone H3 (pHH3) expression was reduced in the mutant retinas compared to wild-type. (C) Quantification of BrdU incorporation in P0 orJ retinal cells overexpressing nlsGFP (control), VSX2, VSX2[R200Q], or VSX2[R227W]. (D) Quantification of BrdU incorporation in P0 wild-type retinal cells overexpressing nlsGFP (control), VSX2, VSX2[R200Q], or VSX2[R227W]. Cells were transfected at 24 hr following initial plating and cultured for an additional 48.0 hr. BrdU was added for the last 4.5 hr of the culture period. Only wild-type VSX2 enhanced proliferation over the control. * P≤0.05; ** P≤0.01 Scale bars: 100 µm. (TIF) [file pgen.1002924.s002.tif]

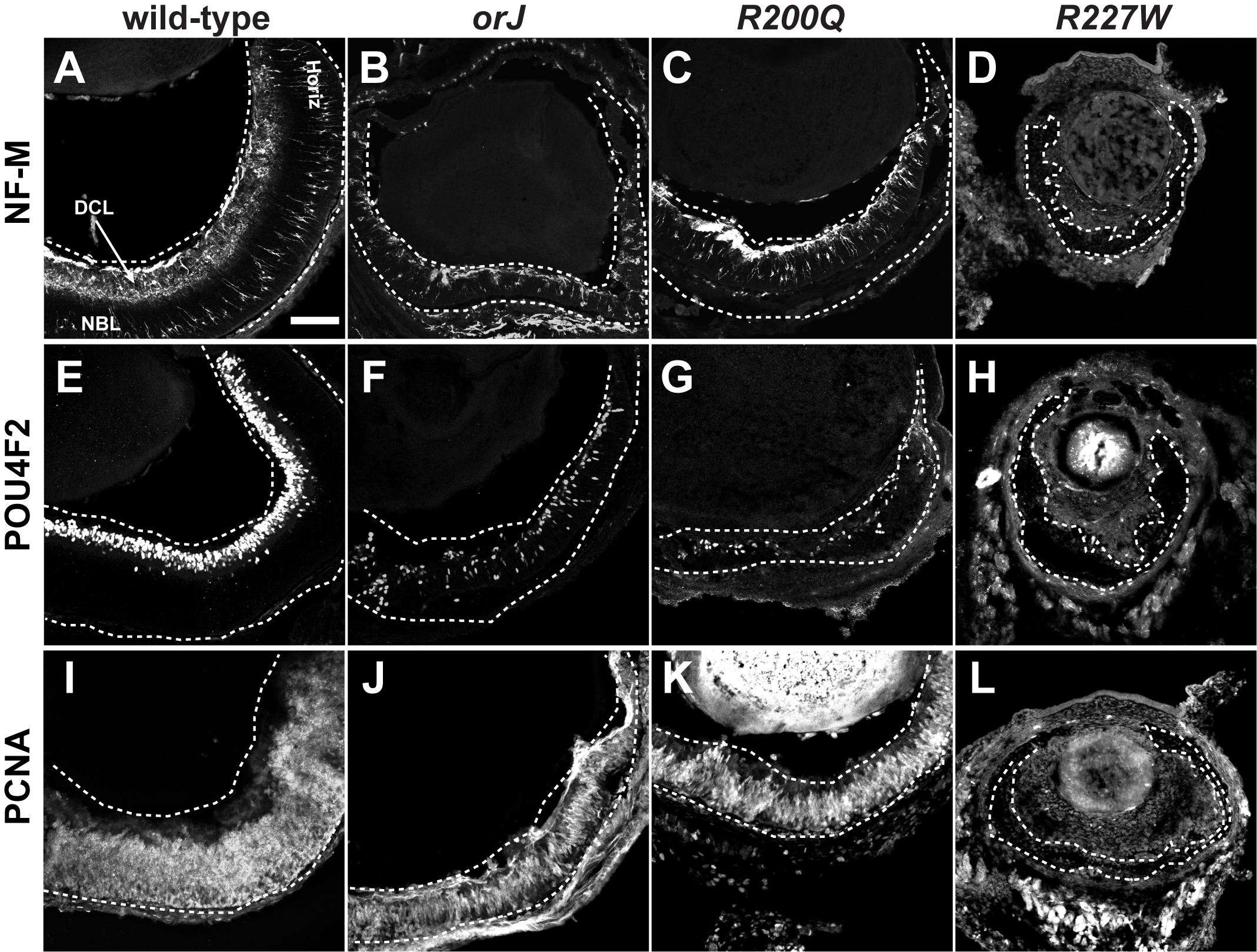

Supplement: Figure S3 — Marker expression in wild-type, orJ, R200Q, and R227W retinas at P0. (A–D) Neurofilament-M (NF-M) expression in inner retinal neurons (differentiated cell layer, DCL) and horizontal cells (horiz), which occupy the neuroblast layer (NBL), was present in all genotypes except R227W. Similar results were obtained for the retinal ganglion cell marker POU4F2 (E–H) and the proliferation/RPC marker PCNA (I–L). Dashed lines bound retinas. Scale bar: 100 µm. (TIF) [file pgen.1002924.s003.tif]

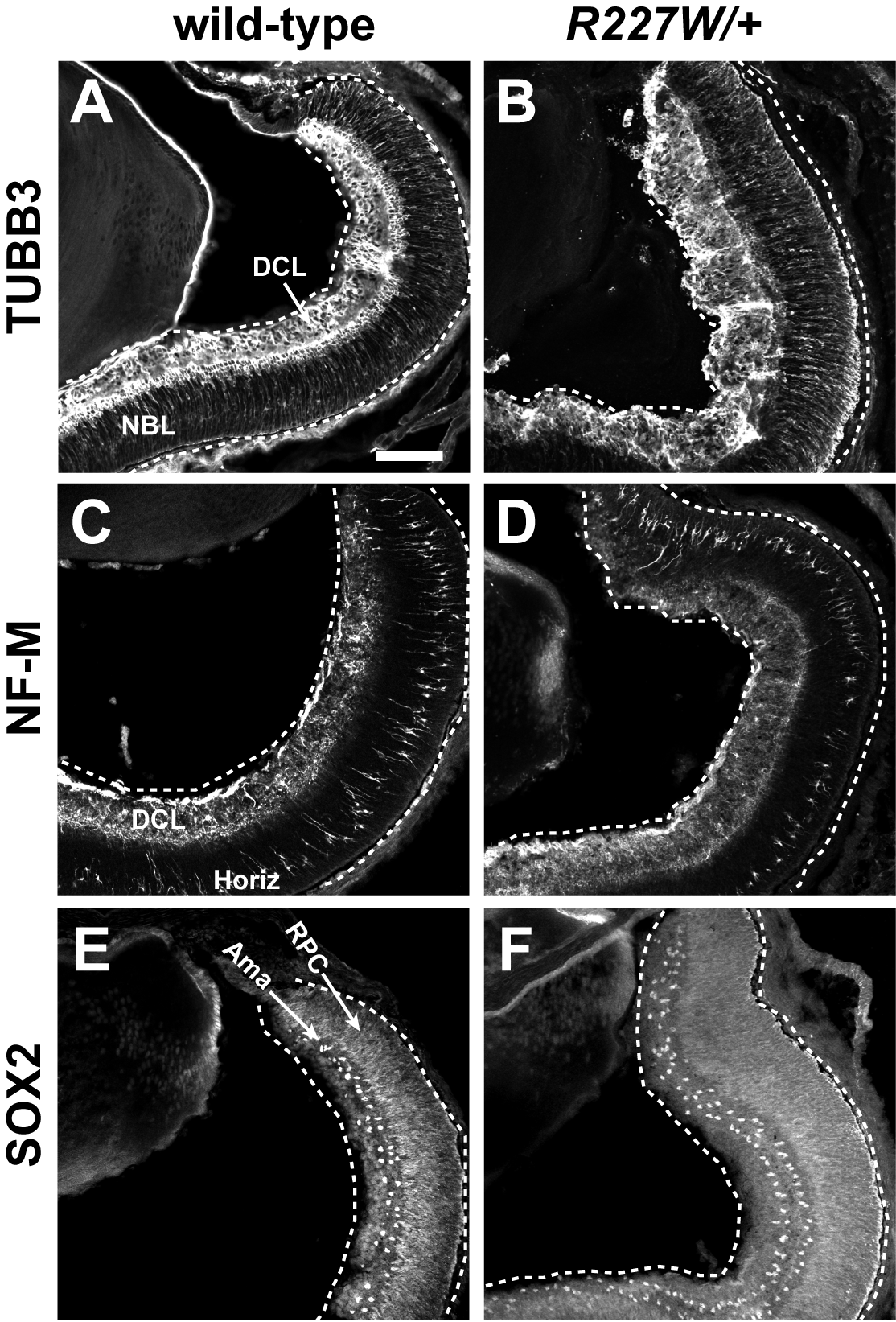

Supplement: Figure S4 — Marker expression in wild-type and R227W/+ retinas at P0. Expression patterns of TUBB3 (A,B), NF-M (C,D), and SOX2 (E,F) in wild-type and R227W/+ retinas were similar. Scale bars: 100 µm. (TIF) [file pgen.1002924.s004.tif]

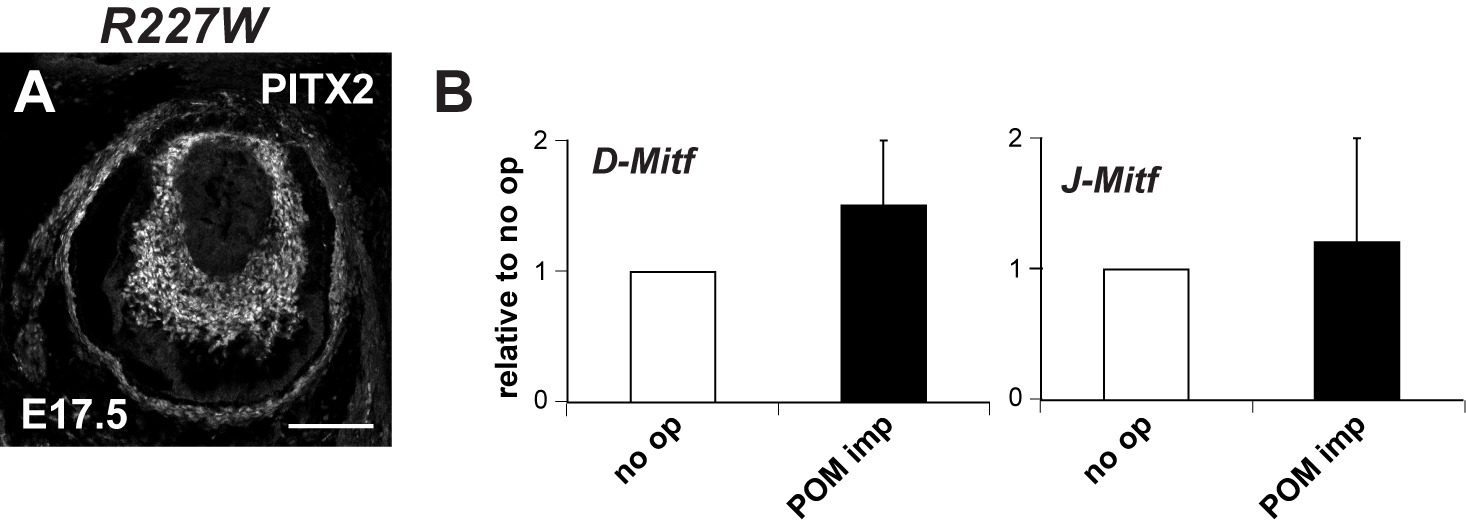

Supplement: Figure S5 — Invasion of POM into the vitreal chamber in R227W eyes. (A) PITX2+ cells filled the vitreal chamber in the E17.5 R227W eye. (B) The relative expression levels of D-Mitf and J-Mitf were not statistically different between control (−POM) and POM implanted whole retina and lens explant (POM imp) cultures (E10.5+2DIV). (TIF) [file pgen.1002924.s005.tif]

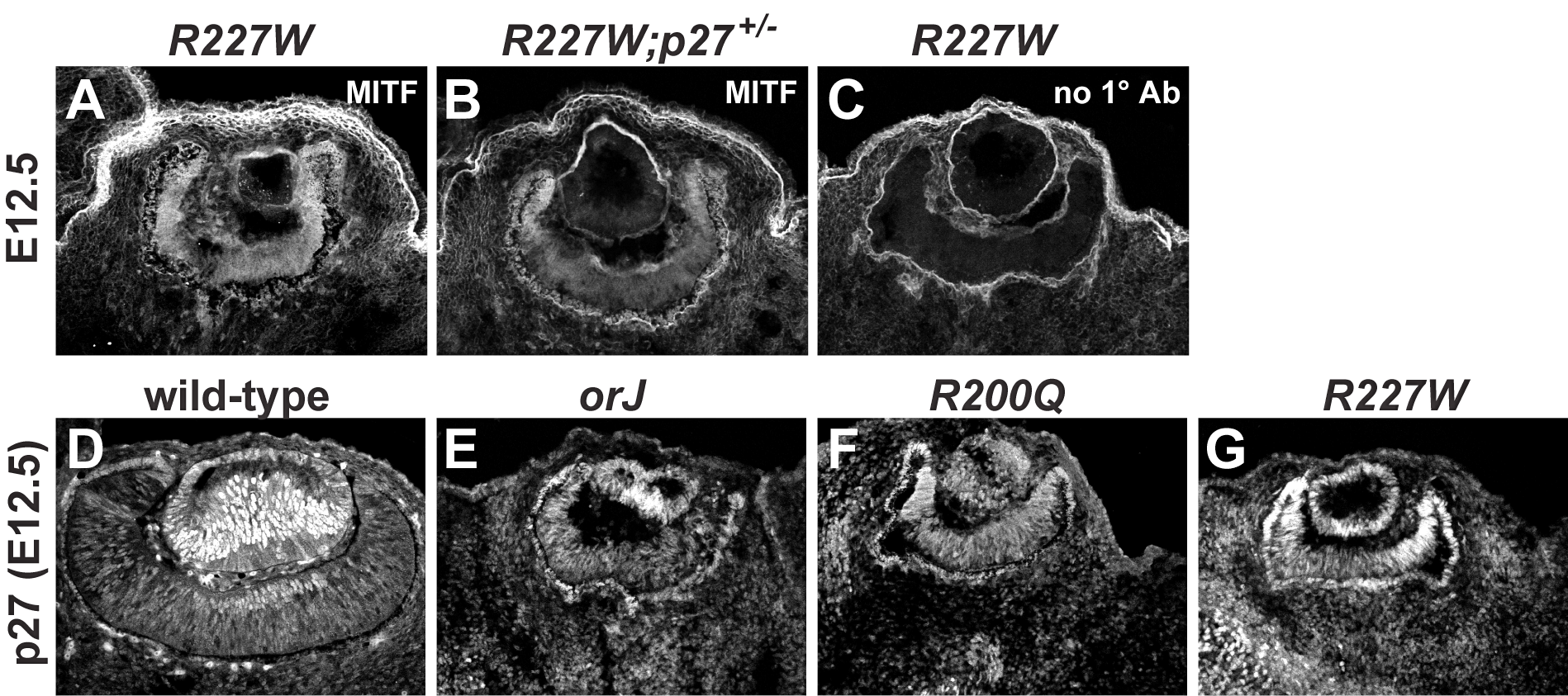

Supplement: Figure S6 — Immunolocalization of MITF and p27 in selected genotypes at E12.5. MITF expression in R227W (A) and R227W; p27+/− (B) eyes. (C) No primary antibody control. p27 expression in wild-type (D), orJ (E), R200Q (F), and R227W (G) eyes. Antigen retrieval was used for p27 staining to reveal staining in neuroblast layers. Bright staining at inner surface of central retina of wild-type is newly generated postmitotic precursors. Bright staining at the periphery of the R200Q retina is also observed occasionally in orJ retina at this age. p27 is also abundantly expressed in lens and surrounding extraocular tissues. (TIF) [file pgen.1002924.s006.tif]
