# Supplementary material for: Vsx2 Controls Eye Organogenesis and Retinal Progenitor Identity Via Homeodomain and Non-Homeodomain Residues Required for High Affinity DNA Binding
Source: PLoS Genet. 2012 Sep 20;8(9):e1002924. doi: 10.1371/journal.pgen.1002924 (PMC3447932; doi:10.1371/journal.pgen.1002924)
Supplement: Table S1 — Oligonucleotides. (DOC) [file pgen.1002924.s007.doc]

**Table S1: Oligonucleotides**

**GENOTYPING**

***R200Q* and *R227W* alleles**

Forward: 5’-ttctggaaccacacctggcatg-3’

Reverse: 5’-ggcaaaatgggccatttgttg-3’

***orJ* allele**

Forward: 5’-AGATGATTATGACCAAGG-3’

Reverse: 5’-CCTGGAGATAGAATCATTTG-3’

***mi* allele**

Forward: 5’-ggtgtgcctcagtcactaatg-3’

Reverse: 5’-ccccaagtcaaatgatccag-3’

***p27* allele**

Forward (wild type): 5’-tggaaccctgtgccatctctat-3’

Forward (mutant): 5’-ccttctatcgccttcttgacg-3’

Reverse (wild type and mutant): 5’-ggttctcatgaactgagactgg-3’

**EMSA**

**P3 element**

plus strand: 5’-gctaatttaattagctagag-3’

minus strand: 5’-ctctagctaattaaattagc-3’

**ChIP**

***D-Mitf* integenic region:**

**Primer set 1 (amplification fragment: -2149 – -2055)**

Forward: 5’-tgtttttgagcttactgttgcctg-3’

Reverse: 5’-gctgtctctactctctacctac-3’

**Primer set 2 (amplification fragment: -2083 – -1920)**

Forward: 5’-gctgtctctactctctacctac-3’

Reverse: 5’-cagtttggactccagattgtcc-3’

**Primer set 3 (amplification fragment: -1941– -1748)**

Forward: 5’-cagtttggactccagattgtcc-3’

Reverse: 5’-ctggtcttacaatgaggtcctg-3’

**Primer set 4 (amplification fragment: -1769 – -1587)**

Forward: 5’-ctggtcttacaatgaggtcctg-3’

Reverse: 5’-ctcactctgaagaccaggctggc-3**’**

**Primer set 5 (amplification fragment: -1609 – -1397)**

Forward: 5’-ctcactctgaagaccaggctggc-3’

Reverse: 5’-cctttaccatcatagaggaggc-3’

**Primer set 6 (amplification fragment: -1418 – -1162)**

Forward: 5’-cctttaccatcatagaggaggc-3’

Reverse: 5’-gagaactcgagtccactctgtg-3’

**Primer set 7 (amplification fragment: -1183 – -1065)**

Forward: 5’-gagaactcgagtccactctgtg-3’

Reverse: 5’-catacttgtggccatgcctgtgg-3’

**Primer set 8 (amplification fragment: -1087 – -842)**

Forward: 5’-catacttgtggccatgcctgtgg-3’

Reverse: 5’-ggattaactagctagagctcaggtt-3’

**Primer set 9 (amplification fragment: -966 – -701)**

Forward: 5’-ttaatctggccagggctttcaccatgg-3’

Reverse: 5’-cccttggaatatcccttacatgaac-3’

**Primer set 10 (amplification fragment: -821 – -677)**

Forward: 5’-ggttcatgggtccaaggatgagc-3’

Reverse: 5’-cccttggaatatcccttacatgaac-3’

**Primer set 11 (amplification fragment: -542 – -231)**

Forward: 5’-ctggtagtcatgtctcacaatgtgg-3’

Reverse: 5’-cctgataagactattatagaagatactt-3’

**Primer set 12 (amplification fragment: -301 – -101)**

Forward: 5’-tgtgtttgttccgttctattgtatagg-3’

Reverse: 5’-cctggtattgcagccagtgacg-3’

**Primer set 13 (amplification fragment: -122 – +11)**

Forward: 5’-cctggtattgcagccagtgacg-3’

Reverse: 5’-ctctccgagaatgttgggacct-3’

***P27^Kip1^*  integenic region:**

**Primer set 1 (amplification fragment: -1099 or -1068 – -883)***

Forward: 5’- GTTGGTAATACCGTGGTGGTGG-3’

Reverse: 5’-GTCTGGACTCAGGTGGAGGGAA-3’

**Primer set 2 (amplification fragment: -927 – -737)**

Forward: 5’-GGAAGGCACTGTTGTAGTTGGC-3’

Reverse: 5’-GCTCTAAAGAGAGGCTTGGGAG-3’

**Primer set 3 (amplification fragment: -758 – -609)**

Forward: 5’-GCTCTAAAGAGAGGCTTGGGAG-3’

Reverse: 5’-GCCTGTCCTACATAGCAGAGAC-3’

**Primer set 4 (amplification fragment: -630 – -438)**

Forward: 5’-GCCTGTCCTACATAGCAGAGAC-3’

Reverse: 5’-CTGAACTAGCCACCGAAGCTCC-3’

**Primer set 5 (amplification fragment: -459 – -196)***

Forward: 5’-CTGAACTAGCCACCGAAGCTCC-3’

Reverse: 5’-CCAACAAACCTGCTCTCTGGCTGG-3’

**Primer set 6 (amplification fragment: -191 – +15)**

Forward: 5’-CGTACACCTCCGAGTAGTCACG-3’

Reverse: 5’-GACTTGCAGTGTCAATCATCTTC-3’

**qRT-PCR**

**pan-*Mitf***

Forward: 5’-gcactctcgagcgtcgtgcatg-3’

Reverse: 5’-GATCGACCTCTACAGCAACCAG-3’

***A-Mitf***

Forward: 5’- TTCTGGTCCAAGTCCCAAGCAG-3’

***B-Mitf***

Forward: 5’-tctgtgcagactcacctcctag-3’

***D-Mitf***

Forward: 5’-gttgggacctgacaggctctga-3’

***H-Mitf***

Forward: 5’-CCTGCTCCTTTGAAAGCTTGTG-3’

***J-Mitf***

Forward: 5’-ctctccatggcagaaggttgac-3’

**All *Mitf* isoforms**

Reverse: 5’-CCAGCCATAAACGTCAGCGTGC-3’

***p27***

Forward: 5’-tttggtggaccaaatgcctgac-3’

Reverse: 5’-CGAACGCTGGCACTGTGGAGCA-3’

***Otx1***

Forward: 5’-GAGCAAGACAAGCCACTCCGAC-3’

Reverse:  5’-cctgaactgctcctggcctca-3’

***Otx2***
Forward: 5’-GCATCAGAGCTGCTGATCTGCC-3’

Reverse:  5’-CTCCAGGCGAATCGAGACCGTC-3’

***Gapdh***

Forward: 5’-gactcatgaccacagtccatgc-3’

Reverse: 5’-gggaaactgtggcgtgatggcc-3’

*: Because of the existence of Homeodomain or/and Mitf putative sites in that region, PCR amplifications for more than one segment was performed.
