# Supplementary material for: Vsx2 Controls Eye Organogenesis and Retinal Progenitor Identity Via Homeodomain and Non-Homeodomain Residues Required for High Affinity DNA Binding
Source: PLoS Genet. 2012 Sep 20;8(9):e1002924. doi: 10.1371/journal.pgen.1002924 (PMC3447932; doi:10.1371/journal.pgen.1002924)
Supplement: Table S2 — Plasmids. (DOC) [file pgen.1002924.s008.doc]

**Table S2: Plasmids**

| **Name** | **Purpose** | **Experiments** | **Vector backbone (Source)** |
| --- | --- | --- | --- |
| pET-26b-VSX2 | In vitro translation | EMSA | pET-26b+ (Novagen) |
| pET-26b-R200Q | In vitro translation | EMSA | pET-26b+ (Novagen) |
| pET-26b-R227W | In vitro translation | EMSA | pET-26b+ (Novagen) |
| pCMV | Empty expression vector (control) | Luciferase assays | pCMV^1^ |
| pCMV-nlsGFP | nlsGFP expression vector (control) | Proliferation assays | pCMV^1^ |
| pCMV-VSX2 | Vsx2 expression vector | Luciferase & Proliferation assays | pCMV^1,2^ |
| pCMV-R200Q | R200Q expression vector | Luciferase & Proliferation assays | pCMV^1,2^ |
| pCMV-R227W | R227W expression vector | Luciferase & Proliferation assays | pCMV^1,2^ |
| pCMV-p27 | p27 expression vector | Luciferase assays | pCMV^1,2^ |
| pCMV-H-Mitf | H-Mitf expression vector | Luciferase assays | pCMV^1,2^ |
| pCMV-H-Mitf(mi) | H-Mitf (mi) expression vector | Luciferase assays | pCMV^1,2^ |
| pCMV-OTX1 | Otx1 expression vector | Luciferase assays | pCMV^1,2^ |
| pGL3P-*DMitf* | Luciferase reporter | Luciferase assays | pGL3P (Promega) |
| pGL3P-*mDMitf* | Luciferase reporter | Luciferase assays | pGL3P (Promega) |
| pGL3B-*DMitf* | Luciferase reporter | Luciferase assays | pGL3B (Promega) |
| pGL3B-*HMitf* | Luciferase reporter | Luciferase assays | pGL3B (Promega) |
| pGL3B-*p27* | Luciferase reporter | Luciferase assays | pGL3B (Promega) |
| pRL-TK | *Renilla* reporter (internal control) | Luciferase assays | pRL-TK(Promega) |
| LexA | Control vector | CAT assays | SVlex (Rod Bremner ref. [34]) |
| LexA-VSX2 | LexA-Vsx2 expression vector | CAT assays | SVlex (Rod Bremner ref. [34]) |
| LexA-R200Q | LexA-R200Q expression vector | CAT assays | SVlex (Rod Bremner ref. [34]) |
| LexA-R227W | LexA-R227W expression vector | CAT assays | SVlex (Rod Bremner ref. [34]) |
| Gal4-HSF1 | Transcriptional activator | CAT assays | Gal4-HSF1 (Rod Bremner ref. [34]) |
| X4G2CAT | CAT reporter | CAT assays | X4G2CAT (Rod Bremner ref. [34]) |

^1^pCMV vector was derived from pEGFP-1 (Clontech) by removal of eGFP cDNA

^2^Kozak consensus sequence cloned into vector backbone
