# Supplementary material for: Vsx2 Controls Eye Organogenesis and Retinal Progenitor Identity Via Homeodomain and Non-Homeodomain Residues Required for High Affinity DNA Binding
Source: PLoS Genet. 2012 Sep 20;8(9):e1002924. doi: 10.1371/journal.pgen.1002924 (PMC3447932; doi:10.1371/journal.pgen.1002924)
Supplement: Table S3 — Primary antibodies. (DOC) [file pgen.1002924.s009.doc]

| **Antibody (host)** | **Target** | **Immunohistology** | **ChIP** | **IP** | **Western blot** | **Source** |
| --- | --- | --- | --- | --- | --- | --- |
| TUBB3 (Rabbit) | ß-Tubulin, class III | 1:4000 |  |  |  | Covance (PRB-435P) |
| TUBB3 (Mouse) | ß-Tubulin, class III | 1:1000 |  |  |  | Covance(clone TUJ1) |
| BrdU (Rat) | BrdU incorporation | 1:10 |  |  |  | Serotec (clone BU1/75) |
| CCND1 (Rabbit) | Cyclin D1 | 1:400 |  |  |  | Lab Vision (RB-212) |
| CCND1 (Mouse) | Cyclin D1 | 1:400 |  |  |  | Santa Cruz (clone 72-13G) |
| MITF(Mouse) | All Mitf isoforms | 1:500 | 1:10 |  | 1:1000 | Exalpha Biologicals (clone C5) |
| OTX (Rabbit) | Otx1, Otx2 | 1:1000 |  |  |  | Chemicon (AB9566) |
| p27 (Mouse) | p27^Kip1^ (Cdkn1b) | 1:500* | 1:10 |  |  | BD biosciences (clone 57) |
| p27 (Rabbit) | p27^Kip1^ (Cdkn1b) | 1:500* |  |  |  | Epitomics (1591-1) |
| pHH3 (Rabbit) | Phosphorylated Histone H3 | 1:500 |  |  |  | Upstate Biotechnology (06-570) |
| PITX2 (Rabbit) | Pitx2 | 1:50 |  |  |  | Tord Hjalt (reference [64]) |
| POU4F2 (Goat ) | Pou4F2 (Brn3b) | 1:50 |  |  |  | Santa Cruz (sc-6026) |
| SOX2 (Rabbit) | Sox2 | 1:400 |  |  |  | Abcam (ab15830) |
| VSX2 (Sheep) | Vsx2, R200Q, R227W | 1:200 | 1:10 | 1:10 | 1:400 | Exalpha Biologicals (X1180P) |

**Table S3: Primary Antibodies**

*requires antigen retrieval for patterns in Figure S6
