# Supplementary material for: Vsx2 Controls Eye Organogenesis and Retinal Progenitor Identity Via Homeodomain and Non-Homeodomain Residues Required for High Affinity DNA Binding
Source: PLoS Genet. 2012 Sep 20;8(9):e1002924. doi: 10.1371/journal.pgen.1002924 (PMC3447932; doi:10.1371/journal.pgen.1002924)
Supplement: Text S1 — Materials and methods for generation of R200Q and R227W knock-in mice. (DOCX) [file pgen.1002924.s010.docx]

**Text S1: Materials and methods for generation of *R200Q* and *R227W* knock-in mice**

The *Vsx2^R200Q^* mutation was generated by targeted gene replacement in embryonic stem (ES) cells. The bases “agg” were changed to “cgc” in exon 4 of the *Vsx2* gene (asterisk) causing the substitution of arginine with glutamine at position 200 of the VSX2 protein. (Figure S1A). The targeting vector was constructed as shown in Figure S1B. The selection cassette ACN [46] was inserted into intron 3 at the XhoI site. The entire DNA was then cloned into pTK1TK2 [88]. The vector was linearized and electroporated into ES cell line TC1 (derived from a 129/SvEvTacfBR mouse) and then selected with G418 and 2'-fluoro-2'-deoxy-1β-D-arabino-furanosyl-5-iodo-uracil. ES cell clones that underwent homologous recombination were identified by southern blot hybridization using the following PCR-generated probes: 5’-*Vsx2* (contained within the recombination region), 3’-*Vsx2* (outside the recombination region) or *neo* (insertion region) following BamHI or HindIII digestion (Figure S1B). ES cells heterozygous for the targeted mutation were microinjected into C57BL/6J blastocysts, generating chimeric mice. Chimeric male mice were then mated with mice from the *orJ* strain (129sv/imj genetic background). *Vsx2^R200Q/+^* or *Vsx2^R200Q/orJ^* mice were intercrossed to generate wild-type, *R200Q* homozygous (*R200Q*) or *orJ* mice. Genotypes were determined by PCR (Figure S1D). ES cell culture, electroporation, and generation of chimeric mice was done at inGenious Targeting Laboratory (Stony Brook, NY).

The *Vsx2^R227W^* mutation was generated by the same procedure except for the following modifications. The bases “agg” were changed to “tgg” in exon 4 of the *Vsx2* gene causing the substitution of arginine with tryptophan at position 227 of the VSX2 protein (Figure S1A). R1 ES cells (129X1/SvJ x 129S1 genetic background; F1 cross) were used for producing gene-targeted cell clones. ES cell culture, electroporation, and generation of chimeric mice was done at the University of Utah Transgenic Core Facility.
